# Synthesis, structure, and magnetic properties of Fe^3+^ and Ru^3+^ metal chalcogenide (O,S) complexes with bidentate ligands

**DOI:** 10.1039/d6dt00055j

**Published:** 2026-03-16

**Authors:** Arsen Raza, José Severiano Carneiro Neto, Arianna Lanza, Jesper Bendix, Matteo Briganti, Lorenzo Sorace, Mauro Perfetti

**Affiliations:** a Department of Chemistry “Ugo Schiff”, DICUS, University of Florence 50019 Sesto Fiorentino Florence Italy mauro.perfetti@unifi.it; b Department of Industrial Engineering, DIEF and INSTM Research Unit, University of Florence 50139 Florence Italy; c Department of Chemistry University of Copenhagen Universitetsparken 5 DK-2100 Copenhagen Denmark; d INSTM Research Unit, University of Florence 50019 Sesto Fiorentino Florence Italy

## Abstract

Hydroxypyridinones represent a versatile class of bidentate ligands for the construction of coordination compounds with tuneable structural and magnetic properties. In this work, we systematically investigate the coordination chemistry and magnetic behaviour of two group VIII trivalent transition metals (Fe^3+^ and Ru^3+^) with 1,2-dimethyl-3-hydroxy-4-pyridinone and its thione analogue, 1,2-dimethyl-3-hydroxy-4-pyridinethione. Tris-chelated octahedral complexes are readily obtained with the oxygen-donor ligand, yielding isostructural compounds stabilized by extended hydrogen-bond networks in the solid state. Substitution of the ketonic oxygen with sulphur markedly alters the reactivity, leading to the formation of a tris-chelated Fe^3+^ complex and an unprecedented sodium-bridged binuclear Ru^3+^ species. Magnetic measurements reveal high-spin (*S* = 5/2) Fe^3+^ behaviour with significant intermolecular antiferromagnetic interactions, while the Ru^3+^ derivatives exhibit a low-spin *S* = 1/2 character. In the sulphur-containing Ru system, the data suggest partial spin delocalization onto the ligand framework. These results elucidate how subtle changes in the donor atom identity and metal electronic structure govern coordination modes, solid-state organization, and magnetic properties, providing valuable insights for the rational design of hydroxypyridinone-based molecular magnetic materials.

## Abstract 2

In this work, we analyse the reactivity and magnetic properties of Fe^3+^ and Ru^3+^ with OPO/OPTO ligands.

## Graphical abstract

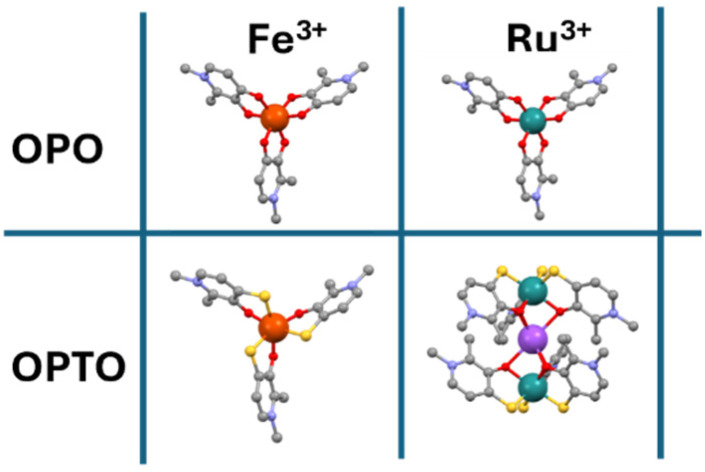

## 1. Introduction

Bidentate ligands are a resource for the coordination of metal ions to generate materials with different topologies. Depending on the bite angle formed at the coordinated atoms, different numbers of chelating ligands can coordinate the same metal ion, generating 1D, 2D and 3D structures. When the metal centre is paramagnetic, such structures can be used to design magnetic molecular materials ranging from single-chain magnets^1^ and single-molecule magnets^2^ to qubits^3^ and magnetic metal–organic frameworks.^4,5^ The engineering of such spin architectures requires a precise knowledge of the magnetic behaviour of the basic building blocks, *i.e.*, the mononuclear metal complexes that can be linked *via* ligands. Desirable characteristics for a versatile ligand platform are the possibility to introduce different substituents and vary the nature of the coordinating atoms.

**Fig. 1:**
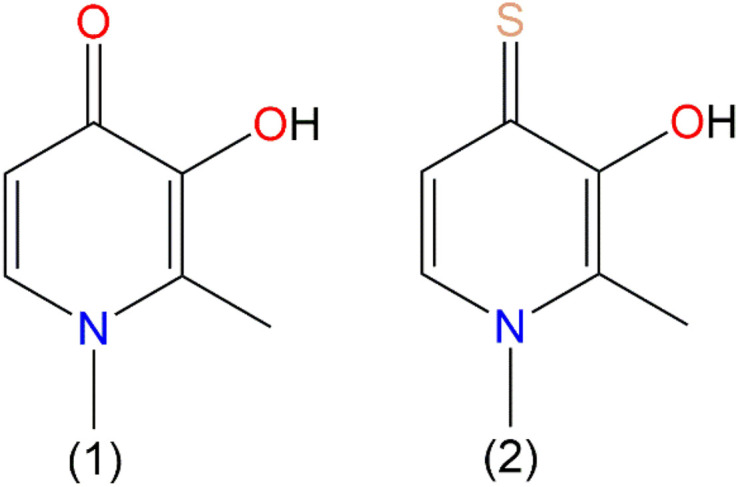
Schematic representation of the ligands 1,2-dimethyl-3-hydroxy-4-pyridinone (1) and 1,2-dimethyl-3-hydroxy-4-pyridinethione (2).

Hydroxypyridinones largely fulfil such requirements. The basic pyridinone structure assures the possibility of coordinating metal ions (especially hard in nature) *via* the oxygen atom. It also provides a simple way to introduce various organic substituents on the N atom of the heterocycle, with 1,2-, 3,2- or 3,4-hydroxypyridinone being all accessible. The solution chemistry of hydroxypyridinones as ligands has been widely explored, mostly by biochemists, in order to target highly stable complexes with metal ions mainly in their +3 ^6–18^ and +4 ^19^ oxidation states. Their high solubility in polar protic environments^20^ prompted particular studies for medical inorganic chemistry purposes. This research focused on *in situ* metal-ion complex formation and subsequent subtraction of metal excess, or the major bioavailability of different metals due to complexation with biologically friendly ligands that can permit a faster and selective absorption in body tissue.^21,22^ In these studies, the Fe^3+^ ion is a standard for exploring the possible applications of hydroxypyridinones as metal-sequestering agents. A clear relationship between the structure of the ligand and the stability of the complex emerged; the 3,4-isomer ensures the highest formation constants of the complex. For this reason, the 3,4-hydroxypyridinone/Fe^3+^ couple is the most studied in the literature. Among all the possible ligands that can be used, the simplest and most widely studied is the anionic form of 1,2-dimethyl-3-hydroxy-4-pyridinone shown in Fig. 1 as 1. For this reason, 1 has been our first choice of ligand.

Comparatively less attention has been given to the properties of the complexes in the solid state. This is especially true for the investigation of coordination chemistry of metal complexes involving hydroxypyridinone ligands and transition metals from the fourth and fifth rows, possibly due to their less predictable chemical reactivity. Additionally, it is noteworthy that modifications to the ligand itself (substitution or addition of functional groups) are limited, with a few examples found in the literature.^23,24^ Among these, one of the most interesting modifications to assess structure–magnetic property relationships is the substitution of the ketone oxygen with another chalcogen. In this work, we focused our attention on extending the study of some group VIII tripositive transition metals with 1,2-dimethyl-3-hydroxy-4-pyridinone (1) and 1,2-dimethyl-3-hydroxy-4-pyridinethione (2). Fe^3+^ and Ru^3+^ possess the same number of d electrons (d^5^), but the increased covalency and spin–orbit coupling lead to significantly different magnetic behaviours.

## 2. Results and discussion

### 2.1. Synthesis and crystal structure

We describe here the synthesis and the crystal structure of the compounds studied in this work. Standard chemical characterization, including IR, NMR, UV-Vis, single-crystal XRD, and PXRD, and unconventional analysis such as electron diffraction are reported in the SI.

**Fig. 2:**
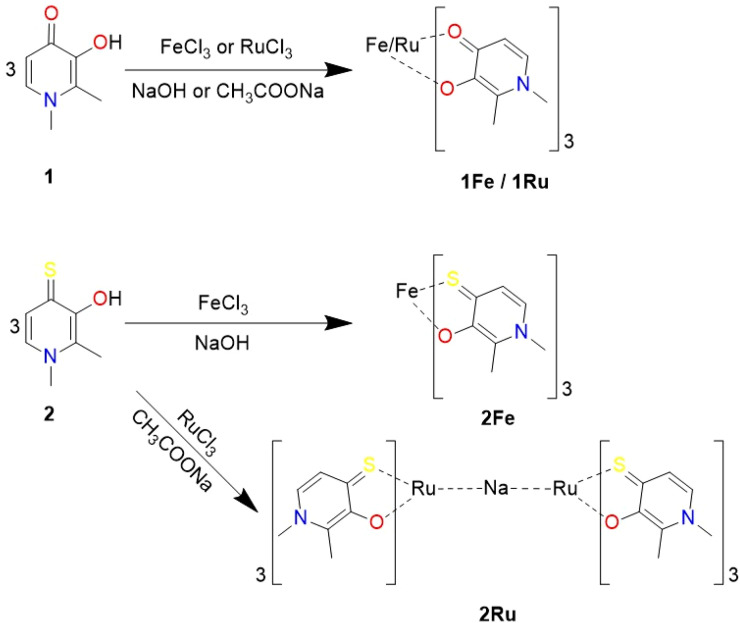
Scheme 1: Principal reactions of 1 and 2 with Fe^3+^ and Ru^3+^.

In Fig. 2, a schematic representation of the major results obtained is reported.

#### 2.1.1 Ligands

While 1 is commercially available (see Fig. S1 for IR), 2 was obtained with a slight modification of a previously reported method.^24^ Under nitrogen, 1 (2.0001 g, 14.374 mmol) and Lawesson's reagent (C_14_H_14_O_2_P_2_S_4_, 2.9003 g, 7.17 mmol) were added in a 250 mL flask. Anhydrous and deoxygenated toluene (≈100 mL) was cannulated in the flask forming a yellow suspension, and stirred under reflux for 4 h. The suspension turns to a solution and then to a viscous oil. The solvent was removed under vacuum, the products were solubilized in hot MeOH (≈70 mL) and a pale-yellow insoluble powder was filtered off. A yellow powder was obtained from the methanolic solution at −20 °C and it was filtered and washed with cold methanol and diethyl ether, affording 0.9312 g of 2 (yield: 41.85%). The IR/ATR spectrum is presented in Fig. S1. EA. calcd for C_7_H_9_OSN (155.22): C, 54.2; H, 5.8; N, 9.0; S, 20.6. Found: C, 53.8; H, 5.9; N, 8.6; S, 20.6. ^1^H-NMR (400 MHz, DMSO-d_6_): *δ* = 8.73 (s, 1 H), 7.66 (d, *J* = 7.4 Hz, 1 H), 7.28 (d, *J* = 6.6 Hz, 1 H), 3.82 (s, 3 H), 2.41 (s, 3 H) ppm.

#### 2.1.2 Tris(1,2-dimethyl-3-hydroxy-4-pyridinonato)iron(iii)·12H_2_O, 1Fe

The complex was synthesized by following a previously reported procedure^7^ with minimal variations. In a flask containing 25 mL of deionized H_2_O and 1 (0.2003 g, 1.439 mmol), 0.7 mL of 2 M solution of NaOH (1.4 mmol) was added dropwise under stirring. The suspension was left stirring until complete solubilization was achieved. 5 mL of a yellow solution of FeCl_3_·6H_2_O (0.1300 g, 0.4809 mmol) was added dropwise and rapidly a red solution starts to form. The solution was refluxed under N_2_ for 1 h, the volume was reduced to half and the solution was left to crystallize at 10 °C. After 24 h, the non-reacted ligand crystallizes and it was removed by filtration; subsequently, keeping the solution at 4 °C for one week gives red prismatic crystals suitable for X-ray analysis. The crystals were collected by suction filtration on a glass frit and gently washed with diethyl ether to give 0.1921 g of 1Fe (yield: 58.34%). IR/ATR, UV-Vis and X-ray crystallography analyses confirm the nature and stoichiometry of the product, see Fig. S1–S3. EA. calcd for Fe(C_7_H_8_O_2_N)_3_·12H_2_O (686.46): C, 36.7; H, 7.0; N, 6.1. Found: C, 36.9; H, 7.0; N, 6.3.

**Fig. 3:**
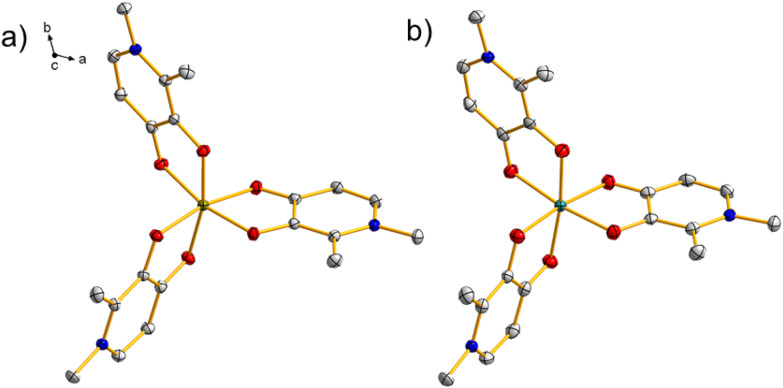
Structures of (a) 1Fe and (b) 1Ru; thermal ellipsoids are drawn at the 50% probability level. Color code: gray = C, red = O, blue = N, brown = Fe, and aquamarine = Ru. The water molecules and H atoms were removed for clarity.

1Fe crystallizes in the trigonal *P*3̄ space-group with two molecules in the unit cell related by an inversion center. The asymmetric unit is composed of one-third of the molecule (*i.e.*, the *C*_3_ axis passes through the complex) and four water molecules. Selected bond lengths and angles are listed in Tables S1 and S2 in the SI. The coordination geometry is pseudo-octahedral (Fig. 3). The lattice water molecules form a network of hydrogen bonds, which extends to the carbonyl oxygen (see Fig. S4). The hydrogen bonds connecting two molecules placed on the same *C*_3_ axis are much shorter than the other ones, favoring the possibility of magnetic interactions. The extension of the hydrogen bond network shows a hexagonal symmetry around six H_2_O molecules that are connected by hydrogen bonds to six different 1Fe molecules. For the 1Fe complex, the relative contributions of intermolecular interactions are illustrated in Fig. S5.

#### 2.1.3 Tris(1,2-dimethyl-3-hydroxy-4-pyridinonato)ruthenium(iii)·12H_2_O, 1Ru

Using the same synthetic procedure to obtain 1Fe and substituting FeCl_3_·6H_2_O with RuCl_3_·6H_2_O did not yield any product that could be characterized. Therefore, we adapted a method previously reported to obtain a similar compound Ru(ma)_3_ (ma = maltolate).^25^ In a flask, 25 mL of EtOH, 1 (0.1023 g, 0.7352 mmol) and CH_3_COONa (0.0976 g, 1.20 mmol) were added. The suspension was stirred until all the ligand was solubilized (10 min). 10 mL of an ethanolic brown solution of RuCl_3_·3H_2_O (0.0332 g, 0.127 mmol) was added dropwise. The initially brown solution was refluxed until it turns deep red (24 h). The solvent was removed under vacuum and a brown solid was obtained. Flash column chromatography CH_2_Cl_2_ : MeOH (1 : 1) was performed and the red band was collected (r.f. 0.2). The solvent was removed under vacuum, the brown-red solid was solubilized in deionized H_2_O and allowed to crystallize at 4 °C. Red crystals suitable for X-ray analysis were obtained after 1 week. The solution was gently filtered, and the crystals were washed with diethyl ether to provide 0.0115 g of 1Ru (yield: 12.38%). IR/ATR and UV–Vis–NIR data for the product are provided in Fig. S1–S3. EA. calcd for Ru(C_7_H_8_O_2_N)_3_·12H_2_O (731.68): C, 34.5; H, 6.6; N, 5.7. Found: C, 34.2; H, 6.3; N, 5.9.

1Ru is isomorphic and isostructural with 1Fe. *i.e.*, the central Ru^3+^ ion is coordinated by three deprotonated ligand molecules, with a similar hydrogen bonding network Fig. S6 and S7. For the 1Ru complex, the relative contributions of intermolecular interactions are illustrated in Fig. S8, which depicts the Hirshfeld surface of the complexes in these highly hydrated neutral complexes. The most significant interactions correspond to H⋯ (49.8%), O⋯H (25.3%), and C⋯H (20.7%). Since the ligand coordinates exclusively through its *O*,*O*-donor set, the pyridinic nitrogen remains non-coordinating and available as a weak hydrogen-bond acceptor, although its contribution to the overall interaction surface is comparatively small, N⋯H (2.5%). The O⋯H bonds arise between water molecules with H (C–CH_3_) and O ligand atoms of neighbouring molecules, occurring at a distance between 1.9150(21) and 2.52547(19) Å. In addition to these, minor intramolecular contacts such as O⋯N (0.8%), N⋯C (0.2%), C⋯ (0.3%) and O⋯C (0.4%) also contribute to the overall surface.

These supramolecular contacts arise naturally from the accessible oxygen atoms and multiple C–H groups in the ligand, producing a packing arrangement governed by a balance of van der Waals interactions and directional H⋯O hydrogen bonds. Together, these interactions stabilize the lattice and explain a similar solid-state organization observed for the 1Fe and 1Ru complexes.

Notably, we also attempted the synthesis of the analogous complex with Os^3+^, but all our attempts failed. However, we managed to synthesize a derivative with one 1 ligand. The failed attempts, as well as the obtained chemical structure and its characterization, are reported in the SI (Fig. S9 and S10 and Tables S3 and S4).

#### 2.1.4 Tris(1,2-dimethyl-3-hydroxy-4-pyridinethionato)iron(iii), 2Fe

The synthesis has been adapted from a literature procedure.^26^ In a flask containing 25 mL of deionized H_2_O and 2 (0.2312 g, 1.489 mmol), 0.75 mL of a 2 M solution of NaOH was added dropwise under stirring. The suspension was left stirring until complete solubilization was achieved. 5 mL of a yellow solution of FeCl_3_·6H_2_O (0.1297 g, 0.4798 mmol) was added dropwise and a dark blue powder starts to precipitate. Under nitrogen, the solution was refluxed for 1 h and, once cooled, the suspension was filtered, affording a dark blue crystalline powder. The powder was washed with H_2_O (150 mL), MeOH (100 mL), acetone (50 mL) and Et_2_O (20 mL) to obtain 0.2063 g of 2Fe (yield: 82.95%). The IR/ATR, UV-Vis and PXRD data for the product are provided in Fig. S1–S3. EA. calcd for Fe(C_7_H_8_OSN)_3_ (518.47): C, 48.6; H, 4.6; N, 8.1; S, 18.6. Found: C, 48.3; H, 4.8; N, 8.1; S, 19.1.

The molecular structure of 2Fe closely resembles that of 1Fe; it is a tris-chelated complex with a crystallographically imposed *C*_3_ symmetry. However, the substitution of the O donor with S hampers the formation of a hydrogen bond network, causing the complex to crystallize without lattice water and having low solubility in any protic solvent. The shortest iron(iii)–iron(iii) distance in the *ab* plane is 9.404 Å, while the shortest distance in the *c*-direction is 7.770 Å.^26^

#### 2.1.5 μ_6_-Sodium-bis(tris(1,2-dimethyl-3-hydroxy-4-pyridinethionato)ruthenium(iii)hydroxide), 2Ru

In a flask, 25 mL of EtOH, 2 (0.1352 g, 0.8710 mmol) and CH_3_COONa (0.1172 g, 1.429 mmol) were added. The suspension was stirred until all the ligand was solubilized (25 min). 10 mL of an ethanolic brown solution of RuCl_3_·3H_2_O (0.0392 g, 0.145 mmol) was added dropwise. The brown solution was refluxed for 24 h. During heating, a dark grey powder starts to precipitate. Once cooled, the solution was filtered and the powder was washed abundantly with H_2_O (150 mL), MeOH (100 mL), acetone (50 mL) and Et_2_O (20 mL) to obtain 0.0262 g of 2Ru (yield: 31.0%). The IR/ATR, UV-Vis and PXRD data for the product are provided in Fig. S1–S3. EA. calcd for Ru_2_(C_7_H_8_NSO)_6_NaOH (1167.40): C, 43.2; H, 4.2; N, 7.2; S, 16.5. Found: C, 43.1; H, 4.2; N, 7.0; S, 16.7.

**Fig. 4:**
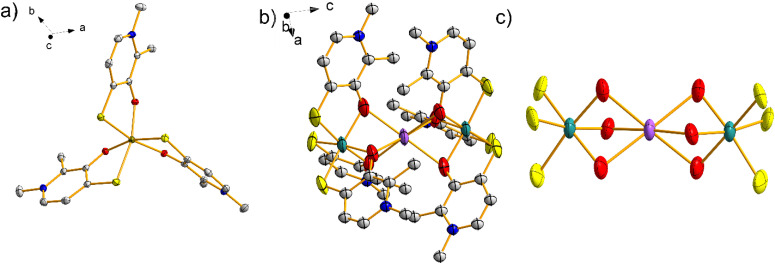
(a) Structure of 2Fe;^26^ thermal ellipsoids are drawn at the 50% probability level. (b) Molecular structure of 2Ru; hydrogen atoms are omitted for clarity. (c) Core of the 2Ru structure with emphasis on the coordinated atoms. Color code: grey = C, red = O, yellow = S, brown = Fe, aquamarine = Ru, and purple = Na.

The extreme insolubility of the grey powder obtained during the synthesis did not allow recrystallization, thus hampering structure determination using X-ray diffractometry. We therefore solved the structure by means of electron diffraction. The obtained structure, solved in the *R*3̄*c* space group, reveals a dimer consisting of two units of the ideal monomer (Ru2_3_), as shown in Fig. S11. Selected bond lengths and angles are given in Tables S5 and S6 in the SI. The two monomers are linked *via* a central Na^+^ ion, which interacts with six oxygen atoms of the ligands of two adjacent monomers (Fig. 4c). A *C*_3_ axis passes through the ruthenium centres, whereas the Na^+^ ion is also sitting on 3 *C*_2_ axes. An OH^−^ anion is present on an inversion centre. Therefore, the asymmetric unit contains 1/3 of the ruthenium atom and 1/6 of Na^+^ and OH^−^. The assignment of the linking unit to Na^+^ has been possible by combining different studies on the complex; elemental analysis using ICP, on the same powder investigated through electron diffraction, revealed a Na : Ru molar ratio of 1 : 1.98, indicating the presence of both sodium and ruthenium in the ratio determined by electron diffraction. On the other hand, the assignment of the excess electronic density external to the dimer to OH^−^ rather than to water is based on the results of the IR-ATR spectrum (Fig. S1). Additional confirmation came from magnetic measurements (*vide infra*).

### 2.2. Magnetic properties and electronic structure

#### 2.2.1 Fe derivatives

The magnetic properties of the synthesized complexes were studied using dc and ac magnetometry on the powder samples. Since the magnetic properties are heavily dependent on the metal ion, we will first discuss the magnetic behaviour and modelling of complexes containing a certain metal ion and then cross-compare between different ions.

**Fig. 5:**
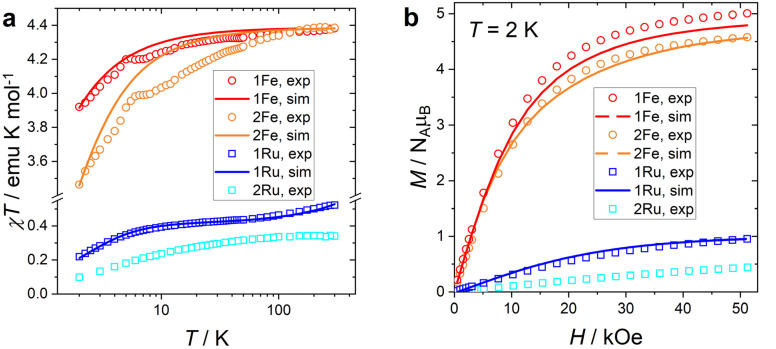
(a) *χT vs. T* product recorded at 1kOe (1Fe and 2Fe) or at 50 kOe (1Ru and 2Ru). (b) Magnetization curves recorded at *T* = 2 K. In both graphs, the symbols represent experimental points and the lines are the best fit (see the text). Data are reported per mole of the metal ion.

The product of the molar magnetic susceptibility times the temperature (*χT*) can be used to assign the spin state of the complexes (Fig. 5a). The room temperature *χT* values of 1Fe and 2Fe are essentially coincident (4.349 and 4.340 emu K mol^−1^) and close to the expected Curie constant for an *S* = 5/2 spin system with isotropic *g* = 2 (*C* = 4.375 emu K mol^−1^). This unambiguously indicates that the Fe complexes are in a high spin state. Upon lowering the temperature, all compounds show a decrease in *χT*, particularly abrupt for the Fe complexes. Such behaviour is commonly attributed either to relevant zero-field splitting or to antiferromagnetic intermolecular interactions. Considering the moderate zero field splitting value expected for high spin Fe^3+^ ions, an orbitally non-degenerate free ion, such an abrupt decrease suggests that intermolecular antiferromagnetic interactions between the molecules are relevant, as expected from our structural analysis. The magnetization curves recorded at *T* = 2 K for the complexes are reported in Fig. 5a. Other temperatures are reported in Fig. S12. The saturation values of 1Fe and 2Fe reach 5 and 4.58*N*_A_*μ*_B_, respectively, close to the expected value of 5*N*_A_*μ*_B_.

**Fig. 6:**
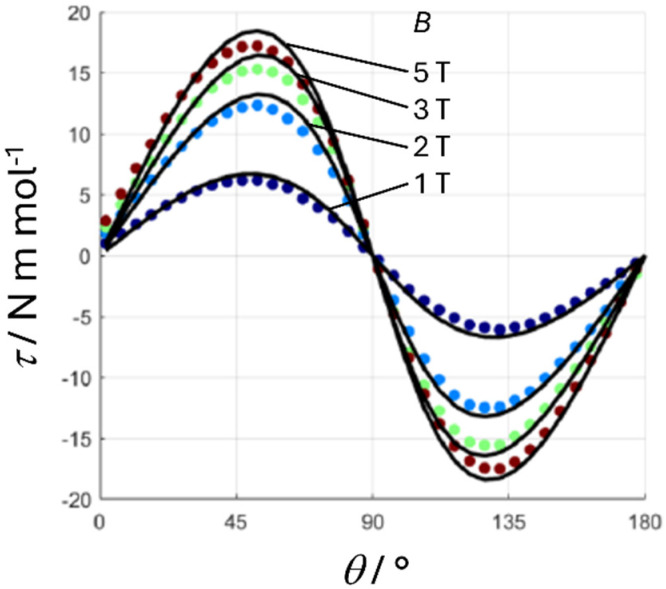
Angular dependence of the magnetic torque of 1Fe measured at *B* = 1, 2, 3, and 5 T (from indigo to wine) at *T* = 2 K. At the beginning of the clockwise rotation around the crystallographic *a* axis, the magnetic field was along the *c* crystallographic axis.

To determine the relevant parameters to describe the electronic structure of the 1Fe complex, we complemented the magnetic measurements with single-crystal torque magnetometry measurements (details about the technique can be found in the SI).^27^ As discussed in the previous section, the unit cell of 1Fe contains two molecules related by an inversion centre, *i.e.*, only one magnetically inequivalent molecule. In Fig. 6, we report a rotation performed along an axis in the *ab*′ plane, thus scanning the axis-to-plane anisotropy of the molecule. The rotation was performed at three different temperatures (5 and 10 K are reported in Fig. S13). As expected from the crystal symmetry, at 0 and 90° (*i.e.* when the *c* axis is parallel and perpendicular to the magnetic field, respectively), the torque is zero. Considering the symmetry of the system,^28,29^ the phase of the torque signal for this rotation is directly related to the nature of magnetic anisotropy, while its magnitude is related to the value of the ZFS parameter *D*. The observed phase of the torque magnetometry clearly points to an easy axis anisotropy and thus to a negative value of *D*.

**Fig. 7:**
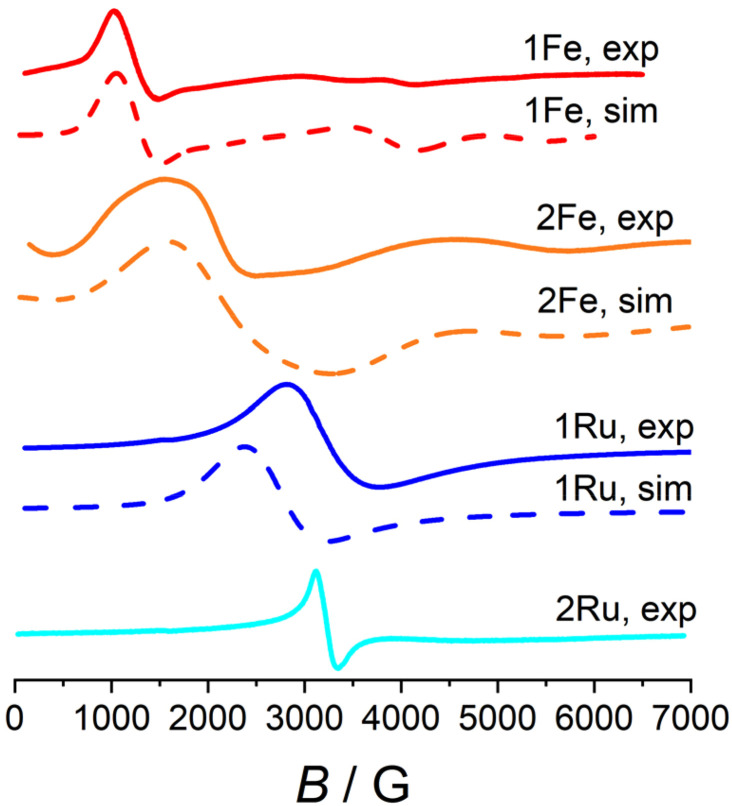
X-band EPR spectra recorded at *T* = 10 K. Continuous lines are experimental spectra and dashed lines are simulations.

To complement the magnetic study, we obtained the X-band EPR spectra of polycrystalline samples of both Fe derivatives at low temperature (see Fig. 7). 1Fe exhibited a strong resonance at 1200 G (*g*_eff_ = 5.6) and other minor features at higher fields, suggesting that the ZFS value of 1Fe is comparable to the X-band frequency.^30,31^ Similarly, 2Fe exhibits a main feature at 2050 G (*g*_eff_ = 3.3) and another weak feature at higher fields. In both cases, the broadness of the peaks supports the existence of diffuse intermolecular magnetic interactions, in agreement with the outcome of dc magnetic measurements.

In order to reproduce all the experimental observables for 1Fe and 2Fe, we considered an axial spin Hamiltonian:^32^1
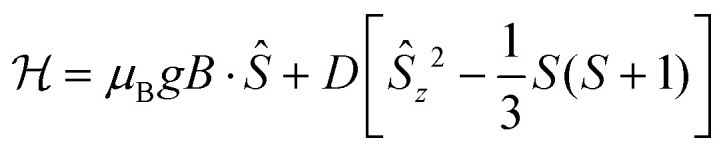
where the first term describes the Zeeman effect and the second one is the zero-field splitting arising from the combined effect of distortion from the octahedral symmetry and spin–orbit coupling.

The best fit of the torque magnetometry data for 1Fe provided *g* = 2 and *D* = −0.32 cm^−1^ (black lines in Fig. 6). The obtained value for *D* is, as qualitatively expected, comparable with the X-band excitation energy (*hν* ≈ 9.4 GHz ≈ 0.31 cm^−1^) and well reproduces the experimental EPR spectrum (dashed red line in Fig. 7). However, these values cannot be used to reproduce the low temperature part of the *χT* plot (Fig. 5) due to the onset of intermolecular interactions. Therefore, we added a mean field correction^33^ to the modelling of magnetic susceptibility:2
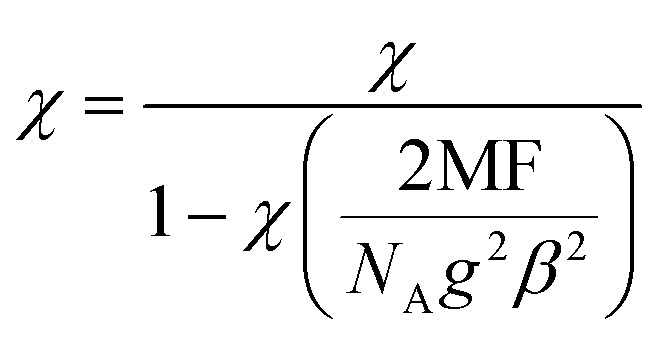
where a negative (positive) value of the MF constant implies AFM (FM) interactions. The best simulation was obtained using MF = −2 × 10^−2^ cm^−1^. The simulation accurately reproduces both the *χT vs. T* and the magnetization data (red solid line in Fig. 5).

Since the poor solubility of 2Fe did not allow to grow crystals of sufficient size to estimate the *D* parameter from torque magnetometry, we employed DFT calculations for estimating its magnitude (see the SI for details), providing *D* = −0.11 cm^−1^. This value provided an excellent starting guess for the subsequent simulation, which followed the model reported for 1Fe. The best results indicate *D* = −0.143 cm^−1^ and MF = −6 × 10^−2^ cm^−1^. This parametrization excellently reproduces the EPR signal (dashed orange line in Fig. 7), the magnetization plot (solid orange line in Fig. 5b), and the *χT* behaviour at high and low temperatures (solid orange line in Fig. 5a). However, the temperature evolution of *χT* is not well reproduced, with a maximum deviation of *ca.* 5% at 10 K. This might be due to the specific crystal packing imposing directionality to the magnetic interactions and causing the mean field model to be less accurate.

#### 2.2.2 Ru derivatives

The room-temperature *χT* value, reported per mole of Ru atoms, of 1Ru and 2Ru (Fig. 5a) are 0.522 and 0.340 emu K mol^−1^, respectively. We notice that while the first value is typical of a *S* = 1/2 system with residual orbital angular momentum, the second value is very close to the one expected for a pure *S* = 1/2 system (*C* = 0.375 emu K mol^−1^). This indicates that the two ruthenium centers in 2Ru are in the +3 oxidation state or that a radical species is formed with concomitant reduction of the metal (*vide infra*). The magnetization saturation value of 1Ru (0.953*N*_A_*μ*_B_) is close to the one expected for a typical *S* = 1/2 system (1*N*_A_*μ*_B_), while the highest magnetic moment recorded for 2Ru is significantly lower (0.442*N*_A_*μ*_B_), suggesting the presence of significant AFM interactions between the two *S* = 1/2 units.

The low-temperature EPR spectra of 1Ru and 2Ru exhibit a single broad peak, compatible with a species of *S* = 1/2 and *g* = 2.10 for both compounds.

The modelling of the Ru encompassing species included several terms in the Hamiltonian, as commonly employed for heavy transition metals:^34^3
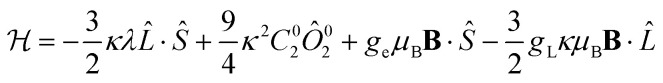


The terms represent the spin–orbit coupling, the crystal field acting on the orbit, the spin and orbital Zeeman terms, respectively. The spin–orbit coupling constant was fixed to the literature value (*λ* = 1180 cm^−1^) and the free electron and orbital g factors were also fixed to the expected values *g*_e_ = 2 and *g*_L_ = 1. The best simulation for 1Ru was obtained using *C*^0^_2_ = 500 cm^−1^ and *κ* = 0.60. Such values suggest a partial reduction of the orbital angular momentum. Interestingly, our best simulation does not accurately reproduce the EPR spectrum, yielding a single feature at *g* = 2.39, while the experimental peak lies at *g* = 2.07. The same issue has been reported by Reynolds *et al.*^35^ for the Ru(acac)_3_ complex. To address this problem, those authors introduced a second set of parameters to model both the low-temperature DC data and the EPR spectrum, which, however, then failed to correctly reproduce the high-temperature data. This behaviour was explained by hypothesizing the onset of a dynamic Jahn–Teller effect affecting the orbital degeneracy of the ^2^T_2g_ ground state, which is blocked at 10 K. In a similar manner, we used a second model with *κ* = 0.34 and *C*^2^_0_ = 1900 cm^−1^, which correctly reproduces the EPR spectrum and the low-temperature DC data (Fig. S14) but does not accurately reproduce the high-temperature data. The resulting orbital reduction factor is significantly smaller than the one obtained with the previous model, while the CF splitting acting on the orbit significantly increases, in consistent with the results provided by Reynolds *et al.*^35^ and supporting the proposed symmetry breaking.

While the model of 1Ru provided a clear picture of its electronic structure and the impact of symmetry on the magnetism, the magnetic properties of 2Ru were extremely hard to rationalise. Using the same Hamiltonian reported in eqn (3) for two metal centres and adding an AFM isotropic exchange interaction term did not provide satisfactorily results. We infer that all the experimental evidence might be consistent if a part of the spin density is actually delocalized on the ligand through a formal reduction of ruthenium(iii) to ruthenium(ii), with the corresponding formation of a radical species on the ligand, as often observed in the Ru complexes.^36^ This non-innocence, that in our complex must be active on both metal centres (formally related by an inversion centre), has been observed in other species of general formula [Ru(acac)_2_(L)]^*n*^ (*n* = −1, 0, +1 and L = redox-active o-quinonoid ligand).^37^ These complexes were described as a combination of two configurations where the spin density is present on the sulphur-donor ligand as a radical species. Interestingly, the X-band EPR measurements carried out on our system at different temperatures are characterized by a signal close to *g* = 2.00, which is observed up to room temperature, a behaviour which is typical of radical species rather than of ruthenium(iii).

## 3. Discussion

**Fig. 8:**
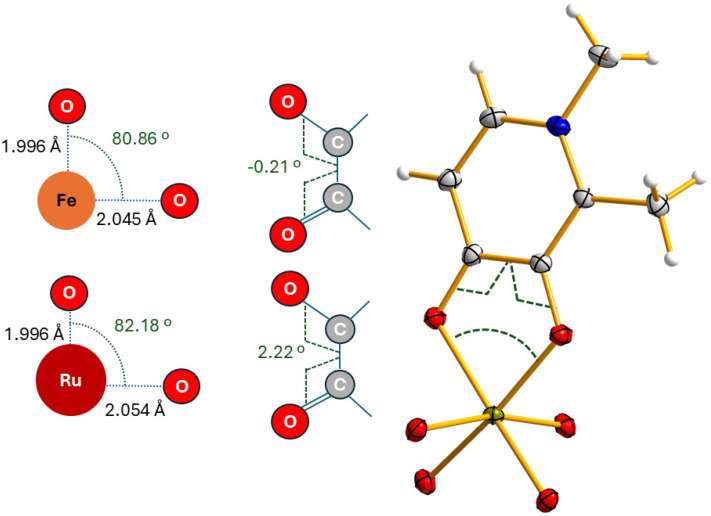
Schematic representation of the O–M–O angles and O–C–C–O torsion angles of 1Fe and 1Ru.

Full chelation of the metal center is achieved, albeit under different conditions, as previously mentioned. It is reasonable to assume that complete chelation for both Fe and Ru is enabled by the effective overlap/combination of the frontier orbitals of the ligand and the metal, which stabilizes the 1Fe or 1Ru complex. The nature of the frontier orbitals for the metals depends, to a good approximation, primarily on their electronic configurations and, of course, on their oxidation states. Using 1Fe, as schematically presented in Fig. 8, as a model, it is possible to define the bond lengths, the coordination bond angle, and the torsion on the ligand. Clearly, the combination of angles and distances represents the most energetically favourable arrangement for the formation of 1Fe, *i.e.*, it allows for optimal overlap of the frontier orbitals. Upon replacing Fe with Ru, the most significant structural difference is the increased torsion of the ligand (2.22° *vs.* −0.21°), which needs to extend (or bend) to reach the frontier orbitals of the Ru centre.

In 1Fe and 1Ru, we observed a significantly more uniform reactivity of the metals with ligand 1, with the complexes being isostructural, despite the markedly different synthetic pathways required to obtain them. While the reactivity of 1Fe has been extensively studied, there are significantly fewer references in the literature regarding 1Ru. For instance, until now, the structure, and consequently the isostructurality with 1Fe, had not been clearly established.

An interesting difference highlighted by our synthetic work is the greater stability of Fe^3+^ compared to that of Ru^3+^ in maintaining its oxidation state when coordinated with ligand 1. During synthesis, the reduction of Ru^3+^ to Ru^2+^ was prevented by using a large excess of ligand under mildly basic conditions. The difference in reactivity between Fe and Ru can be attributed to their different crystal field splitting. 1Fe is a high-spin species, with electrons distributed over both the t_2g_ and e_g_ orbitals, as expected for a weak ligand field. In contrast, 1Ru is a low-spin d^5^ species, due to the large Δ, with partially occupied t_2g_ orbitals. Upon reduction, both complexes would locate the additional electron in the t_2g_ sub shell, driving a larger stabilization for Ru, due to the larger Δ.

Going from ligand 1 to ligand 2, the chemical reactivity becomes more varied, amplified by the distinct softness of sulphur. The 2Fe complex, previously reported, exhibits a plausible and anticipated structure. In contrast, the use of Ru results in markedly different behaviours, at least in the solid state; 2Ru adopts a dinuclear structure composed of two units, each containing one Ru centre and three 2 ligands. The assembly is stabilized by a sodium ion (Na^+^). This species is unexpected, and it is worth noting that the oxygen bridges are favoured thanks to the hard nature of the Na^+^ cation. This newly isolated structure, in some ways unique in its kind, paves the way for a wide range of new compounds to be explored. In fact, it is conceivable to replace the central ion with other cations capable of increasing or decreasing the distance between the two Ru metal centres, potentially enabling the formation of interactions, including tuneable magnetic ones. In our view, the isolation of the dimer is facilitated by a delocalization mechanism where sulphur plays a central role. Such delocalization could also provide an explanation for the atypical magnetic data observed for the dimer.

## 4. Conclusions

This study demonstrates how subtle modifications in ligand donor atoms and metal identity profoundly influence the chemical reactivity and the structural and magnetic properties of hydroxypyridinone-based complexes. The comparison between Fe and Ru reveals that efficient metal–ligand orbital overlap governs the formation and stability of tris-chelated species. The oxygen-to-sulphur substitution in the ligand further diversifies the chemical landscape, yielding unexpected architectures such as the 2Ru dimer, stabilized through µ-oxo bridges. Magnetically, all Fe^3+^ complexes exhibit high-spin behaviour with notable intermolecular antiferromagnetic interactions, while Ru^3+^ derivatives show a low-spin character and, in 2Ru, evidence of partial electron delocalization onto the ligand. Collectively, these results highlight the delicate interplay between ligand flexibility, donor atom identity, and the metal electronic structure in defining both the geometry and spin properties of coordination compounds, providing hints and warnings for the rational synthesis of new molecular materials based on hydroxypyridinone scaffolds.

## Conflicts of interest

There are no conflicts to declare.

## Supplementary Material

DT-055-D6DT00055J-s001

DT-055-D6DT00055J-s002

## Data Availability

The authors confirm that the data supporting the findings of this study are available within the article and its supplementary information (SI). Supplementary information is available. The SI contains chemical characterization (IR, UV-Vis, PXRD, Crystal packing and Hirshfeld potential, crystal structure metrics, additional magnetometric measurements, and failed attempts at obtaining **1Os**. See DOI: https://doi.org/10.1039/d6dt00055j. CCDC 2520137, 2521079, 2520138 and 2520606 contain the supplementary crystallographic data for this paper.^38*a*–*d*^
