# Supplementary material for: Synthesis, structure, and magnetic properties of Fe3+ and Ru3+ metal chalcogenide (O,S) complexes with bidentate ligands
Source: Dalton Trans. 2026 Mar 16;55(21):8119–27. doi: 10.1039/d6dt00055j (PMC13037763; doi:10.1039/d6dt00055j)
Supplement: DT-055-D6DT00055J-s001 [file DT-055-D6DT00055J-s001.pdf]

Supplementary Information (SI) for Dalton Transactions.
This journal is © The Royal Society of Chemistry 2026

Synthesis, structure, and magnetic properties of Fe^{3+} and Ru^{3+} metal chalcogenide (O,S) complexes with bidentate ligands

Arsen Raza[a,b], José Severiano Carneiro Neto[a], Arianna Lanza[c] ,Jesper Bendix[c], Matteo Briganti[a,d], Lorenzo Sorace[a,d], Mauro Perfetti[a,d*]

[a] *Department of Chemistry "Ugo Schiff", DICUS, University of Florence, 50019 Sesto Fiorentino, Florence, Italy;*

[b]*Department of Industrial Engineering, DIEF and INSTM Research Unit, University of Florence, 50139 Florence, Italy;*

[c] *Department of Chemistry University of Copenhagen Universitetsparken 5, DK-2100 Copenhagen, Denmark;*

[d] *INSTM Research Unit, University of Florence, 50019 Sesto Fiorentino, Florence, Italy;*

**mauro.perfetti@unifi.it*

General procedures. Non-anhydrous solvents have been used as received. The precursors $FeCl_3 \cdot 6H_2O$, $RuCl_3 \cdot 3H_2O$, **1** and Lawesson reagent (LW) are commercially available and were used without further purification. $[Os(py)_3Cl_3]$ has been synthesized following a reported procedure.[1] Anhydrous solvents used have been prepared by standard methods.[2] IR spectra were recorded with a Perkin Elmer FT-IR 100 instrument. UV–Vis spectra were recorded on a Jasco V-670 double-beam spectrophotometer by using quartz cuvettes of 1 cm length. ^1H-NMR has been performed on a Bruker AVIII400 UltraShield Plus. EA has been performed with a Thermo Scientific™ FlashSmart™ Elemental Analyzer. ICP-AES was performed in triplicate by a Varian 720-ES inductively coupled plasma atomic emission spectrometer.

X-ray Crystallography. Single-crystal X-ray diffraction data were collected at 100 K using a Bruker D8 Venture diffractometer equipped with a PHOTON II detector and a microfocus source (Cu-Kα radiation, λ = 1.54184 Å). Data were corrected for absorption effects using the Multi-scan method (SADABS). The crystal structure was solved and refined using the Bruker SHELXTL suite.

Powder X-ray diffraction (PXRD) patterns were recorded on a Bruker New D8 Advance DAVINCI diffractometer in a theta-theta configuration equipped with a linear detector. Crystalline powders were mounted on a zero-background diffraction plate. The scans were collected within the range 5–30° (2θ), using Cu Kα radiation.

Electron diffraction crystallography. Measurement performed using the XtaLAB Synergy-ED instrument. The dry powdered sample of **2Ru** was deposited on a 300-mesh copper TEM grid coated with a continuous film of ultrathin amorphous carbon (from EMS). Electron diffraction measurements were performed at ambient temperature and 10^{-5} Pa on a Rigaku XtaLAB Synergy-ED,[3] equipped with a LaB_6 source operating at 200 kV (λ = 0.0251 Å). Series of diffraction patterns were collected with a Rigaku HyPix-ED detector during continuous rotation of the crystals. The program CrysAlisPro was used to control the data collection and process the diffraction data. Datasets were collected from 6 crystals (using a selected area aperture with an apparent diameter of 2μm or 1μm, 0.25° per frame and exposure times of 0.5-1 s per frame) and the structure could be solved from all of them. Finally, the best dataset was selected for the final refinement. The crystal structure was solved ab initio with SHELXT[4] and least-squares refined in kinematic approximation with SHELXL[5] using Olex2[6] and SHELXLe[7], applying the scattering factors for electrons.[8, 9] An extinction coefficient was refined to mitigate the effects of multiple diffraction. Anisotropic displacement parameters were refined for all non-hydrogen atoms. H-atoms were placed in geometrically calculated positions and refined with a riding model, using the C—H distances tabulated for neutron diffraction data.[10]

Magnetic measurements. A Quantum Design Magnetic Properties Measurement magnetometer equipped with a Superconducting Quantum Interference Device Direct was used to perform the DC and

AC magnetic characterization. Temperature- and field-dependent DC magnetization measurements were performed in the 2.0–300 K temperature range on pellets. Experimental results were modeled using the curry function of Easyspin.[11]

EPR spectroscopy. X-band (ν ≅ 9.40 GHz) CW-EPR spectra were recorded on a Bruker Elexsys E500 spectrometer equipped with a ER4122SHQE resonator. Low temperature measurements were obtained using an Oxford Instruments ESR900 continuous flow helium cryostat and temperature controlled by an Oxford Instruments ITC503. The spectrum was simulated with Easyspin.[12]

Torque magnetometry. The cantilever torque measurements were performed using a home made two-legs CuBe cantilever separated by 0.1 mm from a gold plate. The cantilever was inserted in an Oxford Instruments MAGLAB2000 platform with automated rotation of the cantilever chip in a vertical magnet. The capacitance of the cantilever was detected with an Andeen-Hegerling 2500A Ultra Precision Capacitance Bridge. The torque measurements were simulated using a home-written program partially based on EasySpin.

Calculations. All calculations were performed with the ORCA 4.2.1 quantum chemistry package using the PBE0[13] hybrid exchange correlation functional along with D3 atom pairwise dispersion corrections.[14] The X-ray structures were employed. Def2-TZVPP basis sets were utilized for all atoms.[15] The ***D***-tensor was computed within the method proposed by Van Wullen *et al.*[16] while the g-tensor was evaluated within the SOMF(1X) approximation as implemented in ORCA.[17]

Chemical Characterization.

1,2-dimethyl-3-hydroxy-4-pyridinethione, **2.**

UV–Vis–NIR (DMSO): λ max (nm) = 352 nm. IR-ATR (cm^{-1}): 3071 (bm), 1615 (s), 1537 (s), 1493 (w), 1449 (s), 1419 (s), 1376 (w), 1342 (s), 1284 (w), 1266 (s), 1244 (s), 1192 (s), 1169 (m) 1132 (w), 1099 (m), 1047 (w), 1026 (w), 939 (m), 885 (s), 818 (m), 805 (s), 709 (w), 702 (m), 672 (s), 646 (m), 559 (m), 508 (m). ^{1}H-

Tris(1,2-dimethyl-3-hydroxy-4-pyridinonato)Iron(III)·12H_2O, **1Fe**·12H_2O.

UV–Vis–NIR (DMSO): λ max (nm) = 308 nm, 410 nm, 464 nm. IR-ATR (cm^{-1}): 3435 (sh), 3363 (sh), 3193 (s), 2879 (w), 2175 (w), 1606 (m), 1548 (m), 1548 (m), 1505 (m), 1491 (m), 1456 (m), 1428 (w), 1386 (w), 1346 (s), 1303 (m), 1287 (s), 1262 (m), 1175 (w), 1122 (m), 1066 (w), 1034 (m), 916 (w), 823 (m), 763 (m), 704 (m), 619 (w), 577 (w), 552 (w), 537 (w), 537 (w), 495 (w), 476 (w).

Tris(1,2-dimethyl-3-hydroxy-4-pyridinonato)Ruthenium(III)·12H_2O, **1Ru**·12H_2O.

UV–Vis–NIR (DMSO): λ max (nm) = 298 nm, 319 nm, 387 nm, 548 nm. IR-ATR (cm^{-1}): 3374 (vb), 2110 (bw), 1647 (bm), 1486 (s), 1448 (m), 1433 (m), 1381 (w), 1323 (m), 1266 (m), 1168 (w), 1110 (m), 1066(m), 1029 (m), 949 (s), 810 (bm), 767 (bm), 703 (bw), 624 (bw), 578 (bw), 549 (bw), 499 (bw).

Tris(1,2-dimethyl-3-hydroxy-4-pyridinethionato)iron(III), **2Fe**.

UV–Vis–NIR (DMSO): λ max (nm) = 352 nm, 491 nm, 585 nm. IR-ATR (cm^{-1}): 1582 (m), 1531 (w), 1465 (w), 1454 (m), 1434 (m), 1416 (m), 1370 (m), 1287 (m), 1260 (m), 1251 (m), 1192 (m), 1167 (m), 1128(w), 1111 (w), 1050 (m), 1030 (w), 941 (m), 893 (m), 817 (m), 742 (w), 722 (w), 649 (m), 583 (m), 525 (m).

μ_6-sodium-bis(tris(1,2-dimethyl-3-hydroxy-4-pyridinethionato)Ruthenium(III)idroxide, **2Ru**,

UV–Vis–NIR (DMSO): λ max (nm) = 260 nm, 303 nm, 357 nm, 449 nm, 514 nm, 700 nm. IR-ATR (cm^{-1}): 3379 (bw), 1627 (w), 1577 (s), 1439 (s), 1374 (s), 1318 (s), 1274 (s), 1252 (m), 1198 (s), 1169 (m), 1103 (m), 1025 (m), 928 (m), 875 (m), 780 (m), 720 (m), 657 (m), 616 (w), 592 (w), 564 (m), 530 (m), 435 (s).

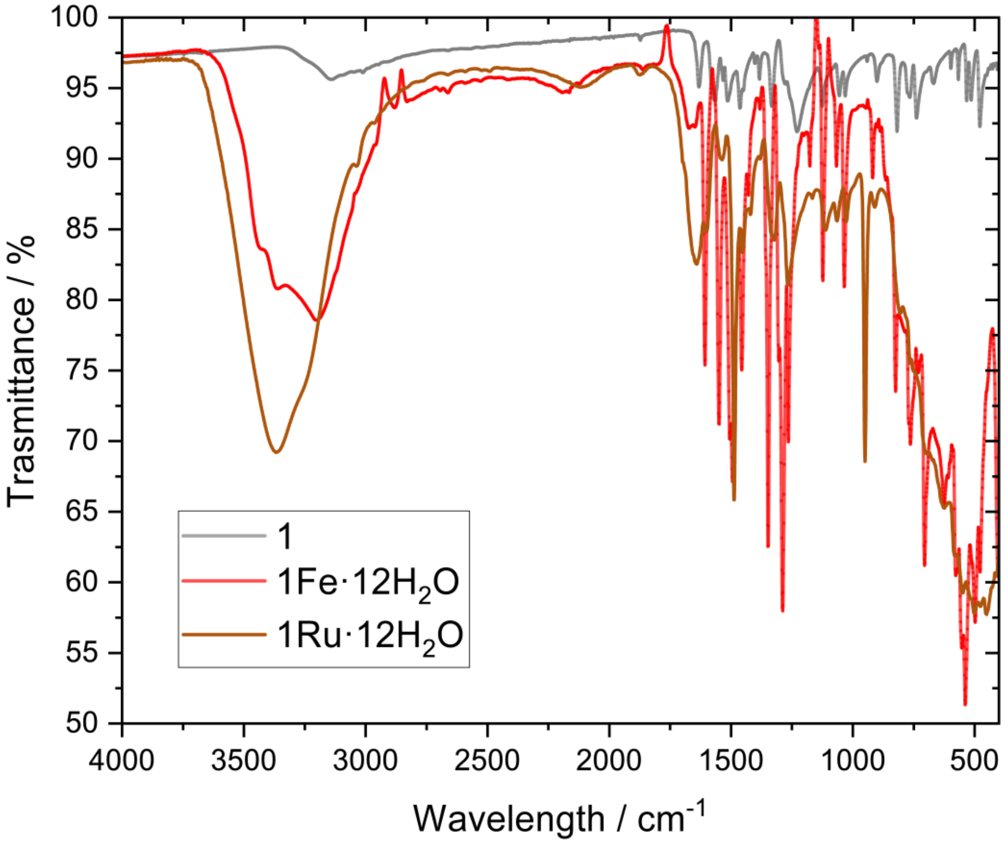

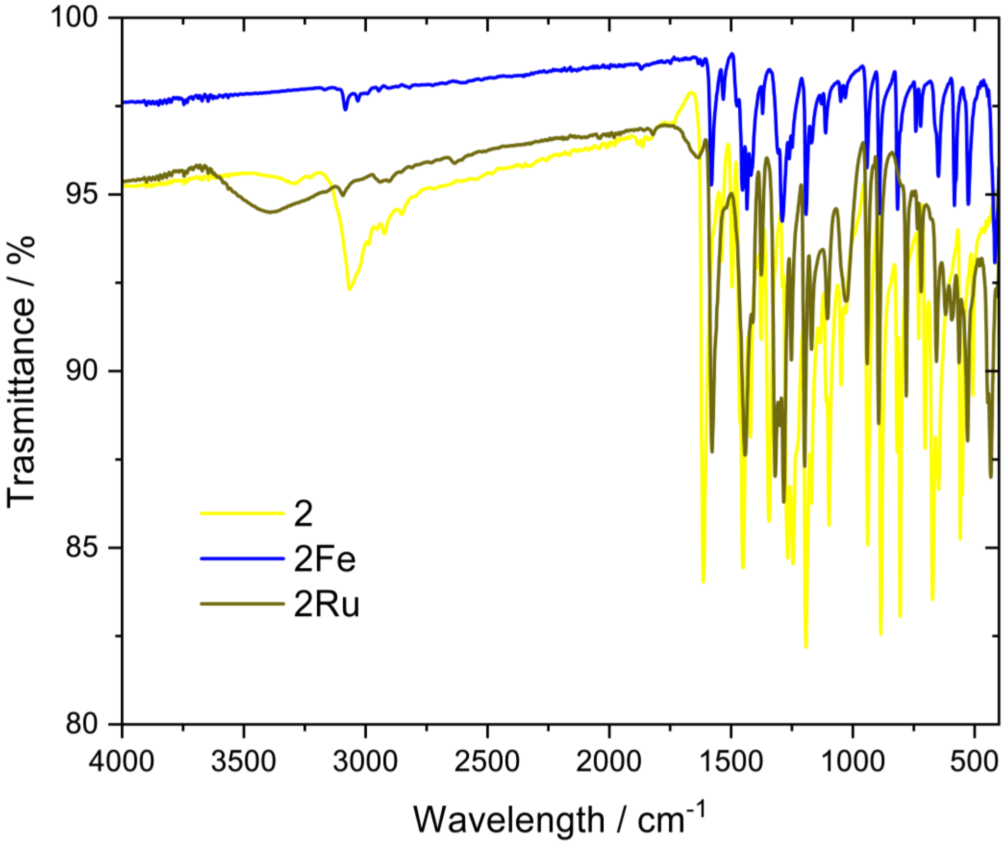

Figure SI-1. IR-ATR spectra recorded for the **1** series (top) and **2** series (bottom).

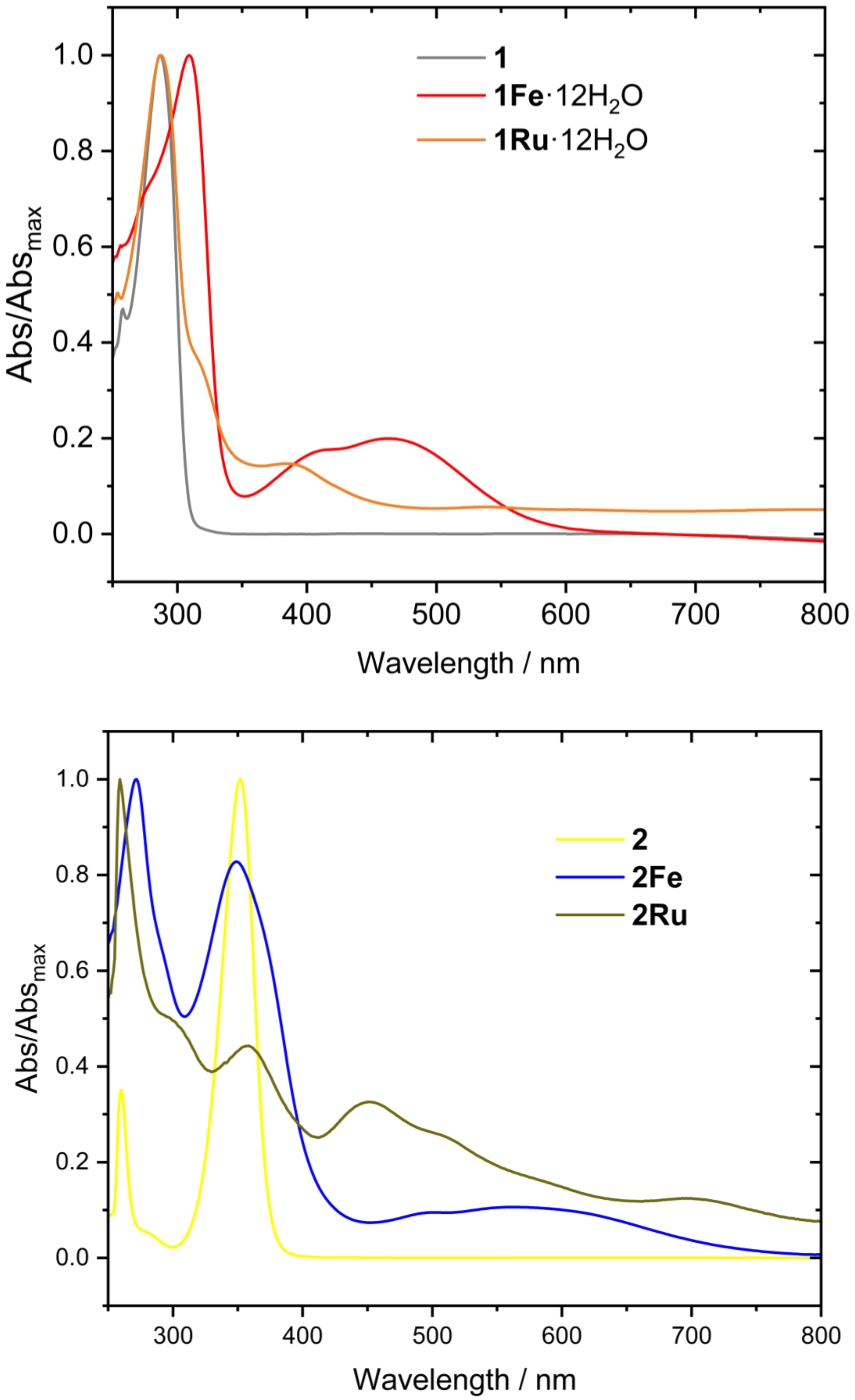

Figure SI-2. UV-Vis spectra recorded in DMSO for the **1** series (top) and **2** series (bottom). All the spectra are normalized on the maximum of absorbance.

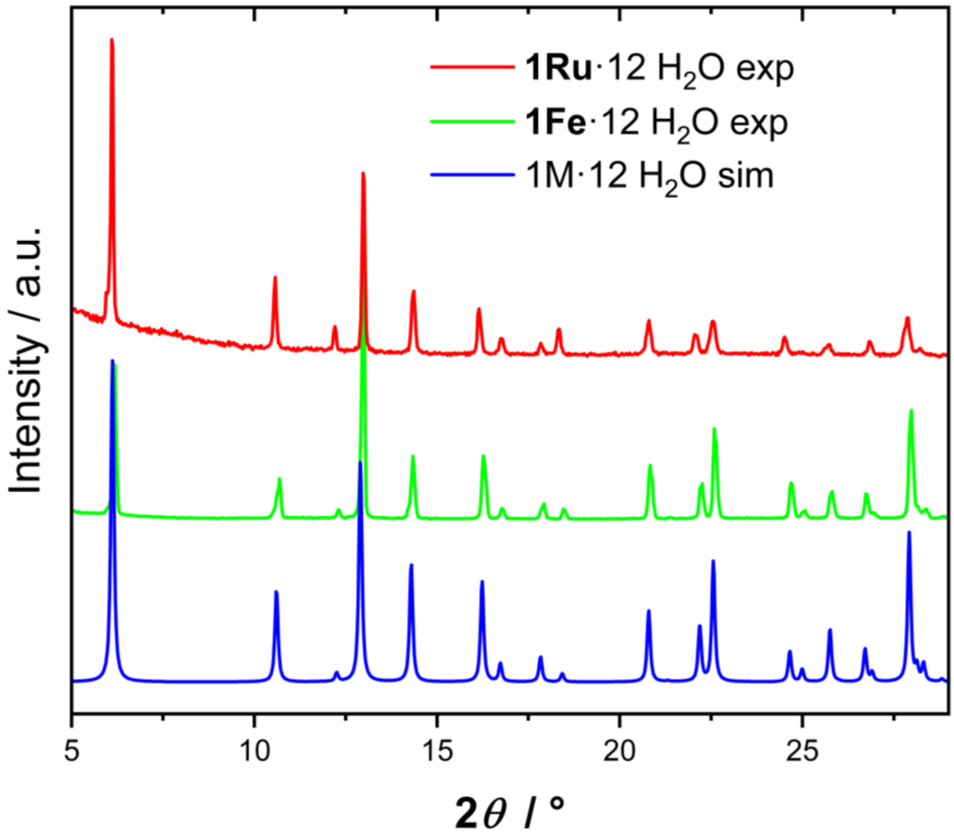

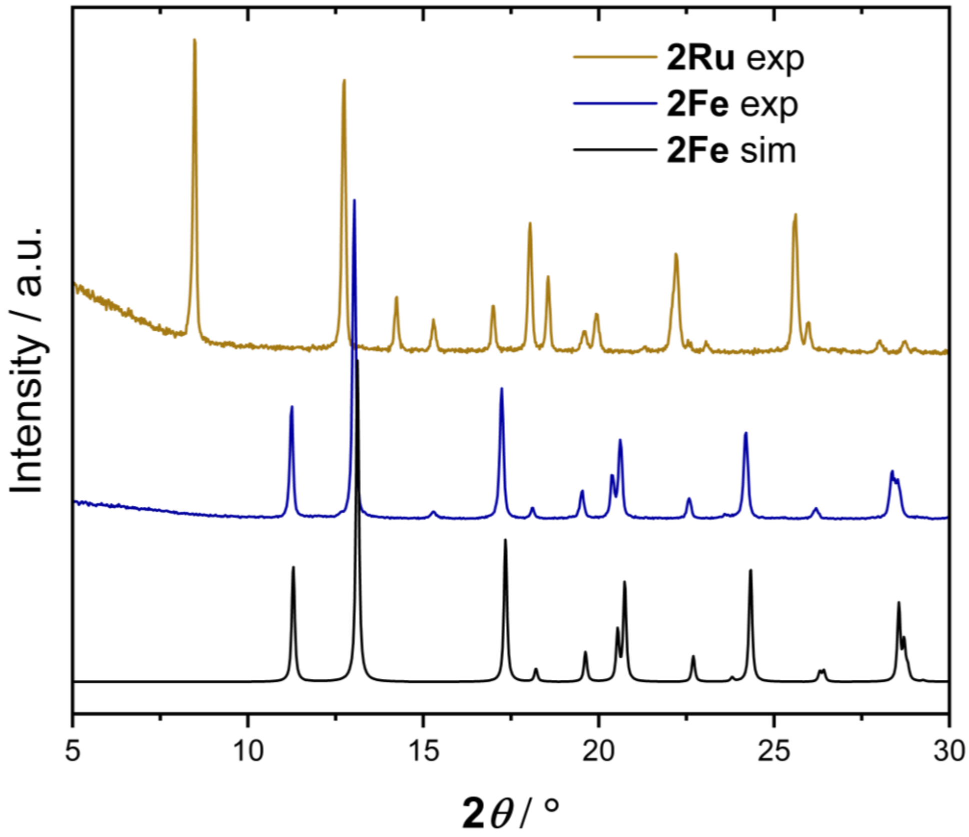

Figure SI-3. Experimental and simulated X-ray powder diffraction pattern for the **1** and **2** species.

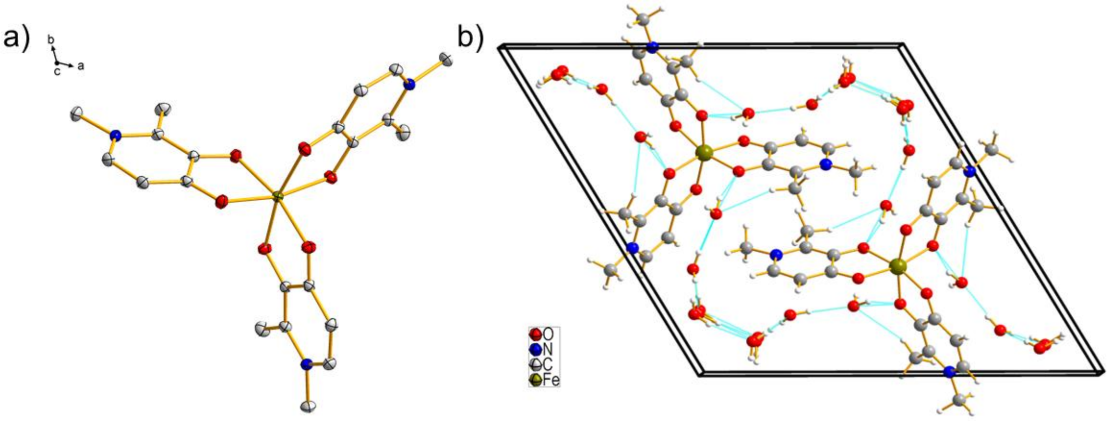

Figure SI-4. a) Structure of **1Fe**, thermal ellipsoids are drawn at the 50% probability level. Color code: gray = C, white = H, red = O, brown = Fe, blue = N. b) View of the unit cell, highlighting the hydrogen bonding interactions.

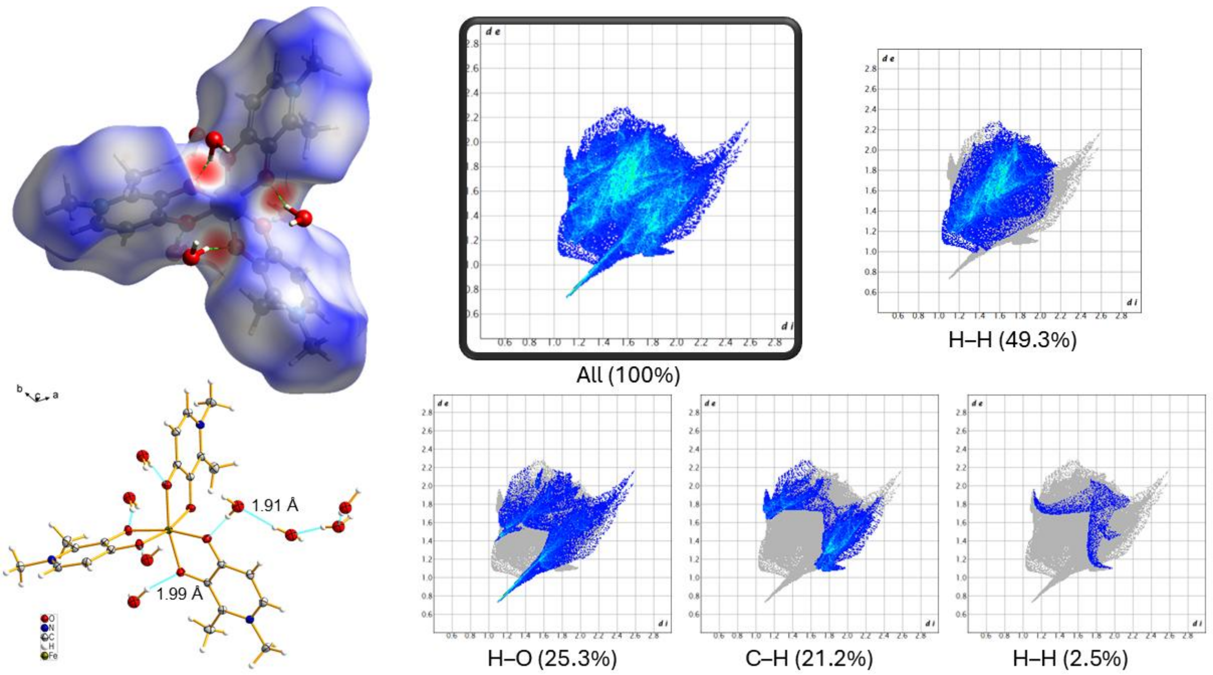

Figure SI-5. Left: Hirshfeld surface for **1Fe**, mapped with d_{norm}, neighbouring water molecules associated with close contacts. Right: Fingerprint plots highlighting the main intermolecular interaction contributions.

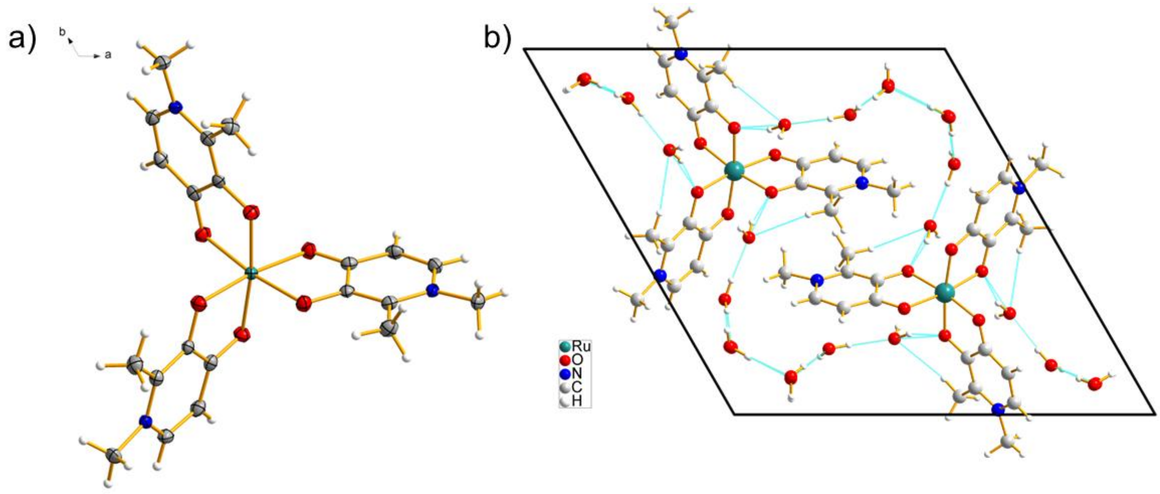

Figure SI-6. a) Structure of **1Ru**, thermal ellipsoids are drawn at the 50% probability level. Color code: gray = C, white = H, red = O, aquamarine = Ru, blue = N. The water molecule is removed for clarity. b) View of the unit cell, highlighting the hydrogen bonding interactions.

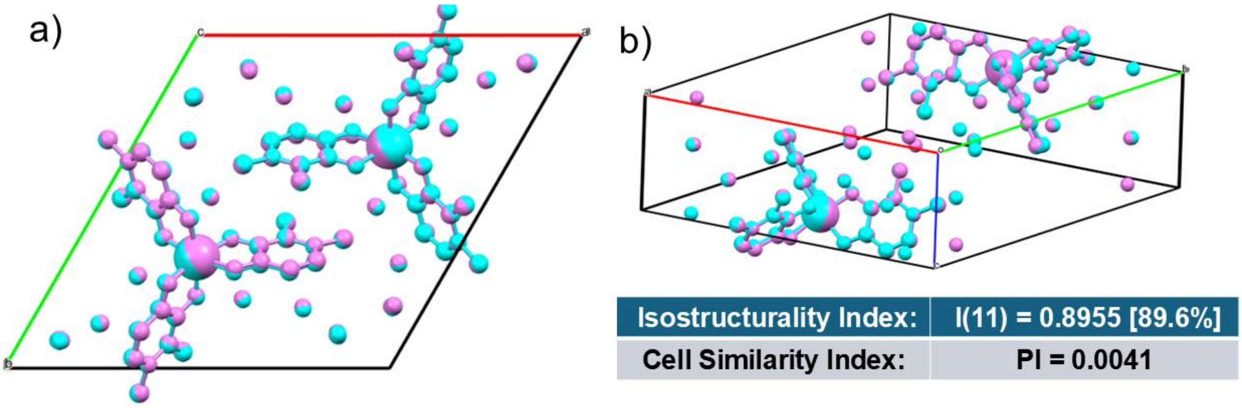

Figure SI-7. a) Structural overlap between **1Fe** and **1Ru**. b) View of the superposition of **1Fe** and **1Ru** unit cell, highlighting the cell similarity values.

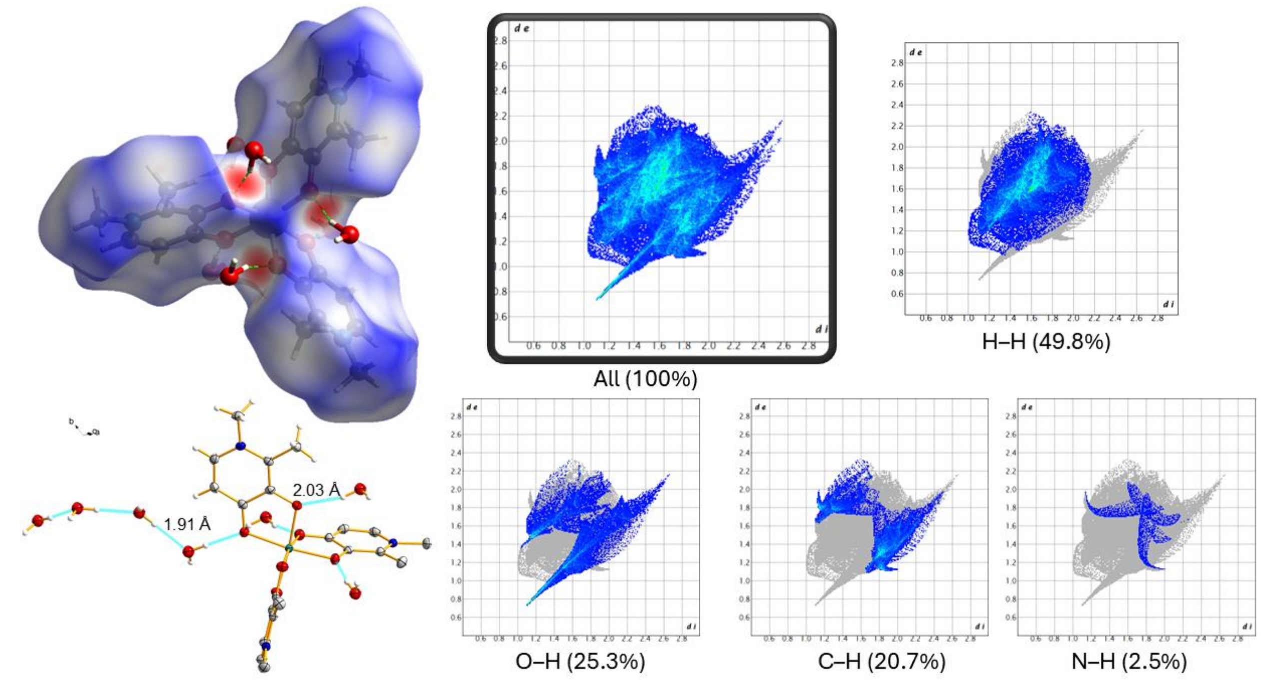

Figure SI-8. Left: Hirshfeld surface for **1Ru**, mapped with d_{norm}, neighbouring water molecules associated with close contacts Right: Fingerprint plots highlighting the main intermolecular interaction contributions.

Table S1. Crystal and refinement data for **1Fe** and **1Ru**, determined with SC-XRD.

	**1Fe**	**1Ru**
Elemental formula	$C_{21}H_{32}FeN_3O_{10}$	$C_{21}H_{32}N_3O_{10}Ru$
CCDC deposition number	2520137	2521079
Formula weight (g mol^{-1})	457.65	243.90
Crystal system / Space group	Trigonal / *P*−3	Trigonal / *P*−3
Temperature (K)	100(2)	100(2)
Unit cell axes (Å)	a = 16.5275(3)<br>b = 16.5275(3)<br>c = 6.7967(1)	a = 16.6350(3)<br>b = 16.6350(3)<br>c = 6.7695(2)
Unit cell angles (°)	α = β = 90<br>γ = 120	α = β = 90<br>γ = 120
Cell volume (Å^3) / Z / F(000)	1607.84(8) /3/730.0	1622.31(7) /6/766.0
Density, calculated (Mg m^{-3})	1.418	1.498
Absorption coefficient (mm^{-1})	0.548	0.562
hkl range	$-27 \leq h \leq 27, -27 \leq k \leq 27, -11 \leq l \leq 11$	$-22 \leq h \leq 22, -22 \leq k \leq 22, -9 \leq l \leq 9$
Reflections collected	55277	48453
Data / restrictions / parameters	4944/0/153	2694/12/156
Goodness-of-fit (F^2)	1.021	1.053
Final R indexes [$I>2\sigma(I)$]	R_1 = 0.0393, wR_2 = 0.1019	R_1 = 0.0312, wR_2 = 0.0753

Final R indexes (all data)	R_1 = 0.0497, wR_2 = 0.1088	R_1 = 0.0344, wR_2 = 0.0765
Largest diff. peak and hole (e Å^{-3})	0.97/-0.48	0.84/-0.69

Table S2. Selected bond lengths (Å) and angles (°)

**Bond length**			
**1Fe**		**1Ru**	
Fe1–O1	1.9961(8)	Ru1–O1^1	2.0537(14)
Fe1–O2	2.0454(8)	Ru1–O1^2	2.0537(14)
O1–C2	1.3320(13)	Ru1–O1	2.0537(14)
N1–C3	1.3694(14)	Ru1–O2	2.0283(14)
N1–C5	1.4703(14)	Ru1–O2^2	2.0282(14)
N1–C6	1.3565(14)	Ru1–O2^1	2.0283(14)
C1–O2	1.3024(13)	O1–C1	1.304(2)
C1–C2	1.4255(14)	O2–C7	1.341(2)
C1–C7	1.4049(15)	N1–C3	1.359(3)
C2–C3	1.3916(14)	N1–C4	1.474(2)
C3–C4	1.4912(15)	N1–C5	1.364(3)
C6–C7	1.3725(16)	C1–C2	1.407(3)
		C1–C7	1.420(3)
		C2–C3	1.364(3)
		C5–C6	1.488(3)
		C5–C7	1.399(3)
**Angles (°)**			
**1Fe**		**1Ru**	
O1–Fe1–O1^1	93.86(3)	O1–Ru1–O1^1	93.16(6)
O1–Fe1–O1^2	91.86(3)	O1–Ru1–O1^2	93.16(6)
O1^1–Fe1–O1^2	91.86(3)	O1^2–Ru1–O1^1	93.16(6)
O1^1–Fe1–O2	167.56(3)	O2^2–Ru1–O1^1	172.68(6)
O1–Fe1–O2^2	167.56(3)	O2–Ru1–O1^2	172.68(6)
O1–Fe1–O2^1	98.41(3)	O2–Ru1–O1	82.19(6)
O1–Fe1–O2	80.86(3)	O2^1–Ru1–O1	172.68(6)
O1^1–Fe1–O2^1	80.86(3)	O2^1–Ru1–O1^2	92.74(6)
O1^2–Fe1–O2	98.41(3)	O2^2–Ru1–O1^2	82.19(6)
O1^2–Fe1–O2^1	167.56(3)	O2^1–Ru1–O1^1	82.18(6)
O1^1–Fe1–O2^2	98.41(3)	O2^2–Ru1–O1	92.74(6)
O1^2–Fe1–O2^2	80.86(3)	O2–Ru1–O1^1	82.74(6)
O2–Fe1–O2^2	90.18(3)	O2^1–Ru1–O2	92.35(6)
O2^2–Fe1–O2^1	90.18(3)	O2^1–Ru1–O2^2	92.35(6)
O2–Fe1–O2^2	90.18(3)	O2–Ru1–O2^2	92.35(6)
C2–O1–Fe1	112.71(6)	C1–O1–Ru1	110.55(12)
C3–N1–C5	120.56(10)	C7–O2–Ru1	109.92(12)
C6–N1–C3	121.33(9)	C3–N1–C4	117.88(17)
C6–N1–C5	118.04(10)	C3–N1–C5	121.14(17)
O2–C1–C2	117.34(9)	C5–N1–C4	120.92(17)
O2–C1–C7	124.52(9)	O1–C1–C2	123.46(18)
C7–C1–C2	118.14(9)	O1–C1–C7	118.01(18)

C1–O2–Fe1	111.87(6)	C2–C1–C7	118.84(18)
O1–C2–C1	116.65(9)	C3–C2–C1	119.84(18)
O1–C2–C3	123.15(9)	N1–C3–C2	121.59(18)
C3–C2–C1	120.19(9)	N1–C5–C6	118.88(17)
N1–C3–C2	119.17(9)	N1–C5–C7	119.42(18)
N1–C3–C4	118.75(9)	C7–C5–C6	121.68(18)
C2–C3–C4	122.07(10)	O2–C7–C1	118.53(18)
N1–C6–C7	121.62(10)	O2–C7–C5	121.55(17)
C6–C7–C1	119.51(10)	C5–C7–C1	119.91(18)

[1]1+y-x,1-x,+z; [2]1-y,+x-y,+z; [1]1-y,1+x-y,+z; 2+y-x,1-x,+z;

Failed attempts at synthetizing **1Os**

As a first attempt, we tried [$(NH_4)_2OsCl_6$] in aqueous solution with the deprotonated ligand in a 1:3 molar ratio. However, the non-innocent redox nature of the ligand, already demonstrated in the synthesis of **1Ru**, did not allow for a clear rationalization of the chemical behaviour of the species formed in solution and produced a mix of compounds that hampered the isolation and characterization of a pure osmium-based compound. Attempts to replicate the synthetic strategies used with ruthenium, leveraging the tendency of **1**-type ligands to stabilize higher oxidation states, also failed. The factors contributing to the lack of expected results with this synthetic strategy are twofold and strongly related: on one hand, the reactivity between **1** and the osmium precursors used, which is different from the one observed with the ruthenium counterpart due to the different redox potential. The second factor is the abundance of accessible oxidation states for osmium that makes its chemistry more complicated and harder to rationalize. As a result of these unsuccessful attempts, we decided to utilize an Os^{III} precursor to simplify the synthetic procedure and gain more reliable insights into the chemical processes taking place. Examples of Os^{III} precursors in the literature are limited primarily to $OsCl_3$. However, attempts to use strategies similar to those applied with the previous precursor proved unsuccessful. Since inorganic precursors were ineffective, we changed our approach and focused on osmium complexes with ligands that could be replaced with **1**. Our first attempt involved [$Os(acac)_3$], but the chelating effect of acac−, combined with the resonating structure of the ligand, stabilizes the complex and renders it inert. Searching through the literature, we selected [$OsCl_3(py)_3$] (py = pyridine) for our purposes, as it is an Os^{III} precursor without a chelating effect to stabilize the complex. The reaction with a chelating ligand like **1** was expected to lead to the substitution of py/Cl− pairs up to a maximum of three times.

The only synthetic procedure which provided reproducible results required using [$Os(py)_3Cl_3$] as starting material. In a double neck flask under nitrogen **1** (0.2520 g, 1.811 mmol) and $NaOCH_3$(0.0920 g, 1.703 mmol), are added to 15 mL of EtOH. The solution is stirred for 1 h. Afterwards, the solvent is removed

under vacuum and 50 mL of anhydrous toluene and solid [$Os(py)_3Cl_3$] (0.2556 g, 0.4787 mmol) are added. The mixture is refluxed for 72 h and then ≈ 80 % of toluene is removed by distillation. The amount of toluene is repristinated, the cycle is repeated 4 times and, at the end, a red solution is obtained. The solvent is removed under vacuum and the compound is purified by chromatography (CH_2Cl_2 : MeOH, 1 : 2) collecting the red band (r.f. 0.4) which upon drying gives 0.0418 g (Yield: 15.66 %) of **Os1(py)$_2$Cl$_2$**. Single crystals suitable for X-Ray diffraction can be obtained by diffusion of Et_2O in a solution of **Os1(py)$_2$Cl$_2$** in CH_2Cl_2.

Os1(py)$_2$Cl$_2$·CH_2Cl_2 crystallizes in the space group $P2_1/c$ with an asymmetric unit consisting of the entire complex molecule and a disordered solvent molecule. **1** imposes on Osmium(III) a bite angle significantly smaller (81.34°) than the one in the precursor $N_{py,\ equat}$–Os–Cl (≈ 89 °). The partial substitution of py with **1** further results in two other significant structural changes around the central metal (see Figure SI-10a). First, the angles Cl–Os–Cl change from 99.55 ° or 90.23 ° (depending on which of the two pairs of Cl^- in *mer*-[$OsCl_3(py)_3$] is considered) to 92.76 ° in **Os1(py)$_2$Cl$_2$**. The fact that the two chlorides are positioned further apart leads to the second structural modification observed in the new complex: the two apical pyridines change their angle relative to the equatorial plane and both tilt toward the ligand **1**. This can be explained by considering two distinct and synergistic contributions: one is the stabilization of the structure by reducing the electrostatic repulsions between chlorides and pyridines, which is expected to be greater than the repulsions between nitrogen and the oxygen of **1**. The second contribution originates from the nature of the ligand **1** and pyridines, which tend to establish π interactions typical of these types of systems. A careful literature review of the various osmium species reveals that the only species with oxygen-donating ligands that enforce a trischelating geometry on a central Osmium(III) is [$Os(acac)_3$][18, 19]. However, it should be noted that in this compound, the bite angle is significantly larger (≈ 93 °) compared to what is expected for the ideal species composed by Osmium(III) and three **1**, which should be in line with other reported species and have a bite angle of approximately 80 °. From the structural analysis conducted so far, it seems reasonable to hypothesize that the subsequent two-step substitution of pyridine and chloride by the ligand, necessary to obtain the desired complex, does not occur due to the combined effect of the ionic size of the metal and the bite angle of the ligand.

Bis(chloride)bis(pyridine)(1,2-dimethyl-3-hydroxy-4-pyridinonato)Osmium(III), **Os1(py)$_2$Cl$_2$**

EA cannot be performed on osmium complexes. UV–Vis (DMSO): λ max (nm) = 258 nm, 279 nm, 363 nm, 392 nm, 523 nm. IR-ATR (cm^{-1}): 3546 (w), 3474 (w), 3104 (w), 3040 (w), 1600 (m), 1539 (m), 1481 (s), 1448 (s), 1388 (w), 1336 (s), 1275 (s), 1211 (m), 1153 (w), 1126 (w), 1067 (w), 1027 (w), 1007 (w), 934 (w), 910 (w), 863 (w), 820 (m), 764 (s), 682 (s), 642 (m), 574 (s), 514 (m), 476 (m).

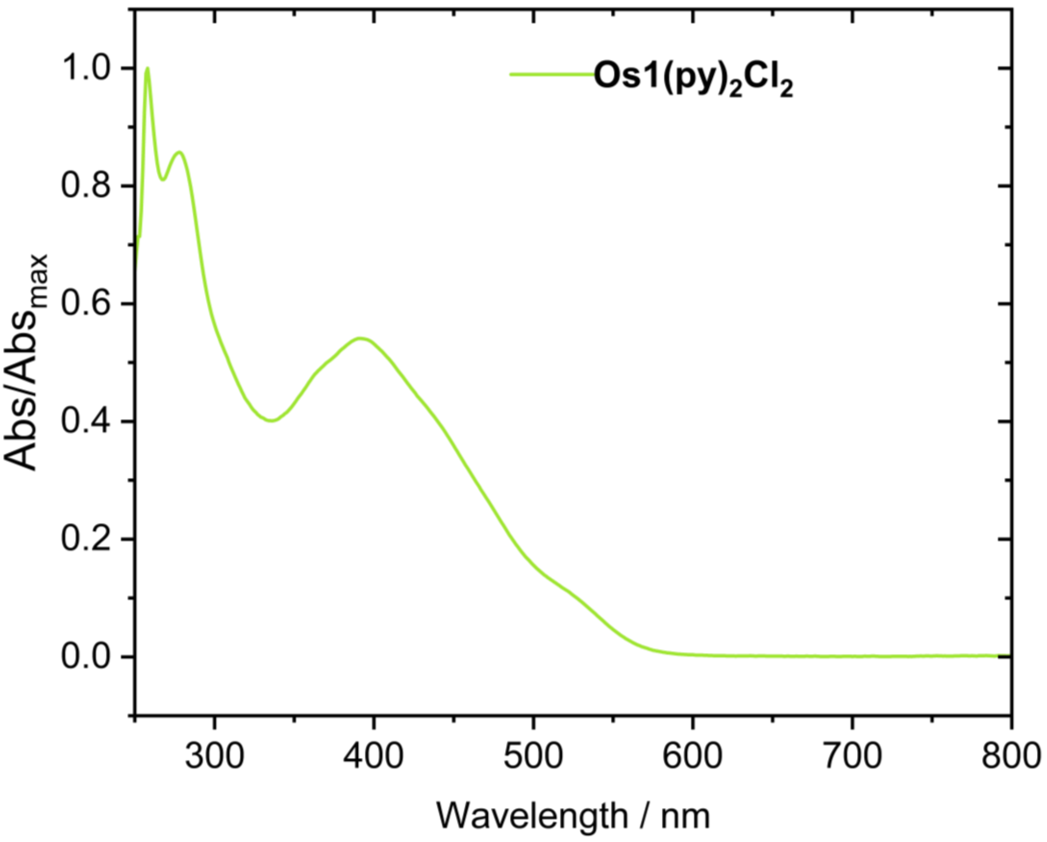

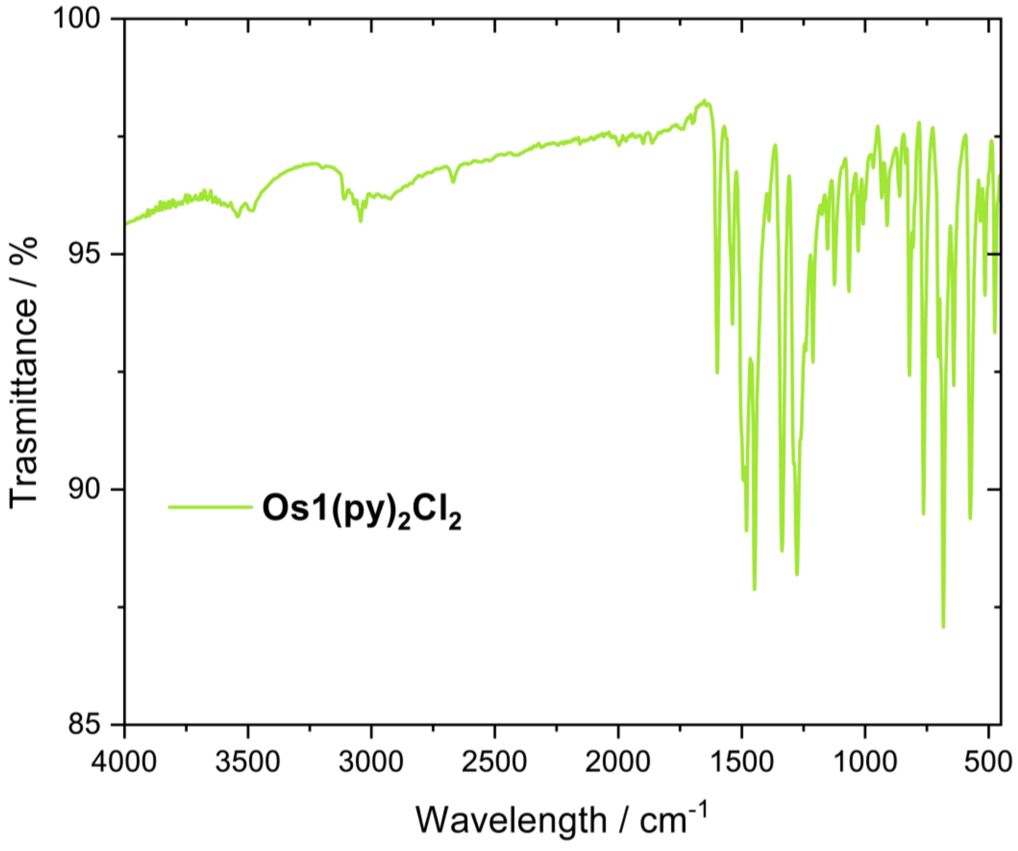

Figure SI-9. UV-Vis and IR-ATR spectra recorded for **Os1(py)$_2$Cl$_2$**.

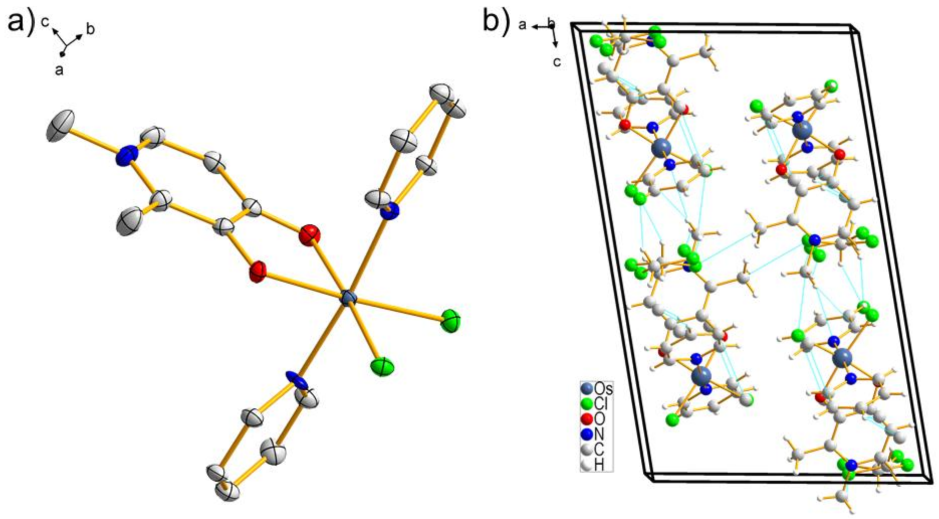

Figure SI-10. a) Structure of **Os1(py)$_2$Cl$_2$**, thermal ellipsoids are drawn at the 50% probability level. Color code: gray = C, white = H, red = O, deep blue = Os, blue = N. The solvent molecules were removed for clarity. b) View of the unit cell with, highlighting the hydrogen bonding interactions.

Table S3. Crystal and refinement data for **Os1(py)$_2$Cl$_2$**.

	**Os1(py)$_2$Cl$_2$**
Elemental formula	$C_{18}H_{19}Cl_4N_3O_2Os$
CCDC deposition number	2520138
Formula weight (g mol^{-1})	641.36
Crystal system / Space group	Monoclinic / $P2_1/c$
Temperature (K)	100(2)
Unit cell axes (Å)	a = 12.3716(7)<br>b = 8.8491(6)<br>c = 20.2098(14)
Unit cell angles (°)	α = γ = 90<br>β = 99.749(2)
Cell volume (Å^3) / Z / F(000)	2180.6(2) /4/1232.0
Density, calculated (Mg m^{-3})	1.954
Absorption coefficient (mm^{-1})	6.357
hkl range	-17 ≤ h ≤ 17, -12 ≤ k ≤ 12, -28 ≤ l ≤ 28
Reflections collected	55707
Data / restrictions / parameters	6707/0/281
Goodness-of-fit (F^2)	1.125
Final R indexes [I>2σ(I)]	R_1 = 0.0396, wR_2 = 0.0974
Final R indexes (all data)	R_1 = 0.0427, wR_2 = 0.0989
Largest diff. peak and hole (e Å^{-3})	2.08/-3.07

Table S4. Selected bond lengths (Å) and angles (°)

$Os1(py)_2Cl_2$			
**Bond length**		**Angles (°)**	
Os1–C11	2.3618(12)	C11–Os1–C12	92.76(4)
Os1–O12	2.3775(12)	O1–Os1–C11	92.88(10)
Os1–O1	2.039(3)	O1–Os1–C12	174.33(10)
Os1–O2	2.059(3)	O1–Os1–O2	81.35(14)
Os1–N1	2.091(4)	O1–Os1–N1	88.22(15)
Os1–N2	2.080(4)	O1–Os1–N2	86.95(15)
O1–C11	1.331(6)	O2–Os1–C11	174.22(11)
Os2–C12	1.300(6)	O2–Os1–C12	93.00(11)
N1–C1	1.340(6)	O2–Os1–N1	88.64(16)
N1–C5	1.340(6)	O2–Os1–N2	87.66(14)
N2–C6	1.342(6)	N1–Os1–C11	91.70(13)
N2–C10	1.340(6)	N1–Os1–C12	92.20(12)
N3–C14	1.343(7)	N2–Os1–C11	91.55(11)
N3–C15	1.491(7)	N2–Os1–C12	92.31(12)
N3–C16	1.376(7)	N1–Os1–N2	174.31(16)
C1–C2	1.377(8)	C11–O1–Os1	110.9(3)
C2–C3	1.390(8)	C12–O2–Os1	111.6(3)
C3–C4	1.399(8)	C1–N1–C5	118.6(4)
C4–C5	1.380(7)	C5–N1–Os1	119.4(3)
C6–C7	1.394(7)	C6–N2–Os1	119.2(3)
C7–C8	1.387(8)	C10–N2–Os1	121.8(3)
C8–C9	1.387(8)	C10–N2–C6	118.9(4)
C9–C10	1.386(7)	C14–N3–C15	118.6(5)
C11–C12	1.435(7)	C14–N3–C16	122.1(5)
C11–C16	1.385(7)	C16–N3–C15	119.3(5)
C12–C13	1.400(7)	N1–C1–C2	121.9(5)
C13–C14	1.375(8)	C1–C2–C3	120.3(5)
C16–C17	1.492(8)	C2–C3–C4	117.6(5)
C13A–C18A	1.765(14)	C5–C4–C3	119.0(5)
C14A–C18A	1.754(14)	N1–C5–C4	122.6(5)
C14B–C18A	1.75(3)	N2–C6–C7	122.5(5)
C18B–C13B	1.71(4)	C8–C7–C6	118.7(5)
		C7–C8–C9	118.7(5)
		C10–C9–C8	120.1(5)
		N2–C10–C9	121.5(5)
		O1–C11–C12	118.2(4)
		O1–C11–C16	121.6(5)
		C16–C11–C12	120.1(5)
		O2–C12–C11	117.8(4)
		O2–C12–C13	123.9(5)
		C13–C12–C11	118.3(4)
		C14–C13–C12	119.4(5)
		N3–C14–C13	121.5(5)
		N3–C16–C11	118.7(5)
		N3–C16–C17	120.5(5)
		C11–C16–C17	120.9(5)

	C14A–C18A–C13A	111.1(8)
	C13B–C18B–C14B	110.1(16)

x, y, z ; −x, y + 1/2, −z + ½; x, −y − 1/2, z − 1/2

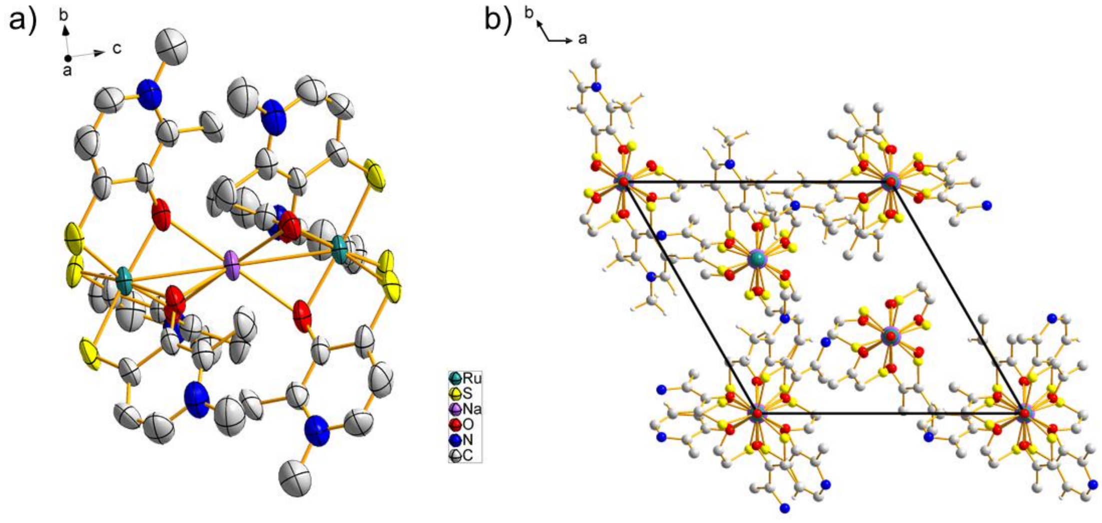

Figure SI-11. a) Structure of **2Ru**, thermal ellipsoids are drawn at the 50% probability level. Color code: gray = C, white = H, red = O, aquamarine = Ru, blue = N. The hydrogen atoms were removed for clarity. b) View of the unit cell showing the molecular packing.

Table S5. Crystal and refinement data for **2Ru** determined with electron diffraction. The cif file was edited and reformatted using the program CIVET.[20]

	**2Ru**
Elemental formula	$C_{42}H_{49}N_6NaO_7Ru_2S_6$
CCDC deposition number	2520606
Formula weight (g mol^{-1})	1167.42
Crystal system / Space group	Trigonal /*R*-3*c*
Temperature (K)	293(2)
Unit cell axes (Å)	a = 13.922(3)<br>b = 13.922(3)<br>c = 41.850(4)
Unit cell angles (°)	α = β = 90<br>γ = 120
Cell volume (Å^3) / Z / F(000)	7025(3)/6/737.0
Density, calculated (Mg m^{-3})	1.656
hkl range	$-14 \leq h \leq 14$, $-16 \leq k \leq 16$, $-48 \leq l \leq 48$
Reflections collected	8760
Data/ restrictions / parameters	1299/0/101
Goodness-of-fit (F^2)	1.116
Final R indexes [I>2σ(I)]	R_1 = 0.1492, wR_2 = 0.4222
Final R indexes (all data)	R_1 = 0.2048, wR_2 = 0.4598

Largest diff. peak and hole (Å^{-2})	0.310 /-0.29

Table S6. Selected bond lengths (Å) and angles (°) of **2Ru**

**2Ru**					
**Bond length**		**Angles (°)**			
Ru1–O1	2.109(10)	O1–Ru1–O1^{1}	86.9(4)	O1^{4}–Na1–O1	104.3(5)
Ru1–O1^{1}	2.109(10)	O1–Ru1–O1^{2}	86.9(4)	O1^{5}–Na1–O1	177.3(5)
Ru1–O1^{2}	2.109(10)	O1^{1}–Ru1–O1^{2}	86.9(4)	O1^{1}–Na1–O1	74.1(4)
Ru1–S1^{2}	2.286(6)	O1–Ru1–S1^{2}	170.4(3)	O1^{2}–Na1–O1	74.1(4)
Ru1–S1^{1}	2.286(6)	O1^{1}–Ru1–S1^{2}	96.2(3)	O1^{3}–Na1–Ru1	135.9(2)
Ru1–S1	2.286(6)	O1^{2}–Ru1–S1^{2}	84.2(3)	O1^{4}–Na1–Ru1	135.9(2)
S1–C1	1.697(14)	O1–Ru1–S1^{1}	96.2(3)	O1^{5}–Na1–Ru1	135.9(2)
Na1–O1^{3}	2.408(10)	O1^{1}–Ru1–S1^{1}	84.2(3)	O1^{1}–Na1–Ru1	44.1(2)
Na1–O1^{4}	2.408(10)	O1^{2}–Ru1–S1^{1}	170.4(3)	O1^{2}–Na1–Ru1	44.1(2)
Na1–O1^{5}	2.408(10)	S1^{1}–Ru1–S1^{2}	93.2(2)	O1–Na1–Ru1	44.1(2)
Na1–O1^{1}	2.408(10)	O1–Ru1–S1	84.2(3)	O1^{3}–Na1–Ru1	44.1(2)
Na1–O1^{2}	2.408(10)	O1^{1}–Ru1–S1	170.4(3)	O1^{4}–Na1–Ru1^{4}	44.1(2)
Na1–O1	2.408(10)	O1^{2}–Ru1–S1	96.2(3)	O1^{5}–Na1–Ru1^{4}	44.1(2)
O1–C2	1.315(15)	S1^{1}–Ru1–S1	93.2(2)	O1^{1}–Na1–Ru1^{4}	135.9(2)
N1–C4	1.353(17)	S1^{2}–Ru1–S1	93.2(3)	O1^{2}–Na1–Ru1^{4}	135.9(2)
N1–C3	1.379(18)	O1–Ru1–Na1	52.6(3)	O1–Na1–Ru1^{4}	135.9(2)
N1–C7	1.357(19)	O1^{1}–Ru1–Na1	52.6(3)	Ru1–Na1–Ru1^{4}	180.0
C1–C5	1.398(17)	O1^{2}–Ru1–Na1	52.6(3)	C2–O1–Ru1	117.7(7)
C1–C2	1.445(14)	S1^{2}–Ru1–Na1	122.98(16)	C2–O1–Na1	136.3(8)
C2–C3	1.399(16)	S1^{1}–Ru1–Na1	122.98(16)	Ru1–O1–Na1	83.4(4)
C3–C6	1.492(15)	S1–Ru1–Na1	122.98(16)	C4–N1–C3	120.7(12)
C4–C5	1.332(18)	C1–S1–Ru1	99.2(4)	C4–N1–C7	118.2(14)
		O1^{3}–Na1–O1^{4}	74.1(4)	C3–N1–C7	121.0(12)
		O1^{3}–Na1–O1^{5}	74.1(4)	C5–C1–C2	115.1(11
		O1^{4}–Na1–O1^{5}	74.1(4)	C5–C1–S1	125.0(9)
		O1^{3}–Na1–O1^{1}	177.3(5)	C2–C1–S1	119.9(10)
		O1^{4}–Na1–O1^{1}	107.7(5)	O1–C2–C3	120.0(10)
		O1^{5}–Na1–O1^{1}	104.3(5)	O1–C2–C1	119.0(11)
		O1^{3}–Na1–O1^{2}	104.3(5)	C3–C2–C1	121.0(12)
		O1^{4}–Na1–O1^{2}	177.3(5)	N1–C3–C2	118.6(10)
		O1^{5}–Na1–O1^{2}	107.7(5)	C2–C3–C6	120.6(12)
		O1^{1}–Na1–O1^{2}	74.1(4)	C5–C4–N1	121.7(13)
		O1^{3}–Na1–O1	107.7(5)	C4–C5–C1	122.8(12)

^{1}1+y-x,1-x,+z; ^{2}1-y,+x-y,+z; ^{3}4/3-x,2/3-x+y,7/6-z; ^{4}1/3+y,-1/3+x,7/6-z; ^{5}1/3-y+x,2/3-y,7/6-z

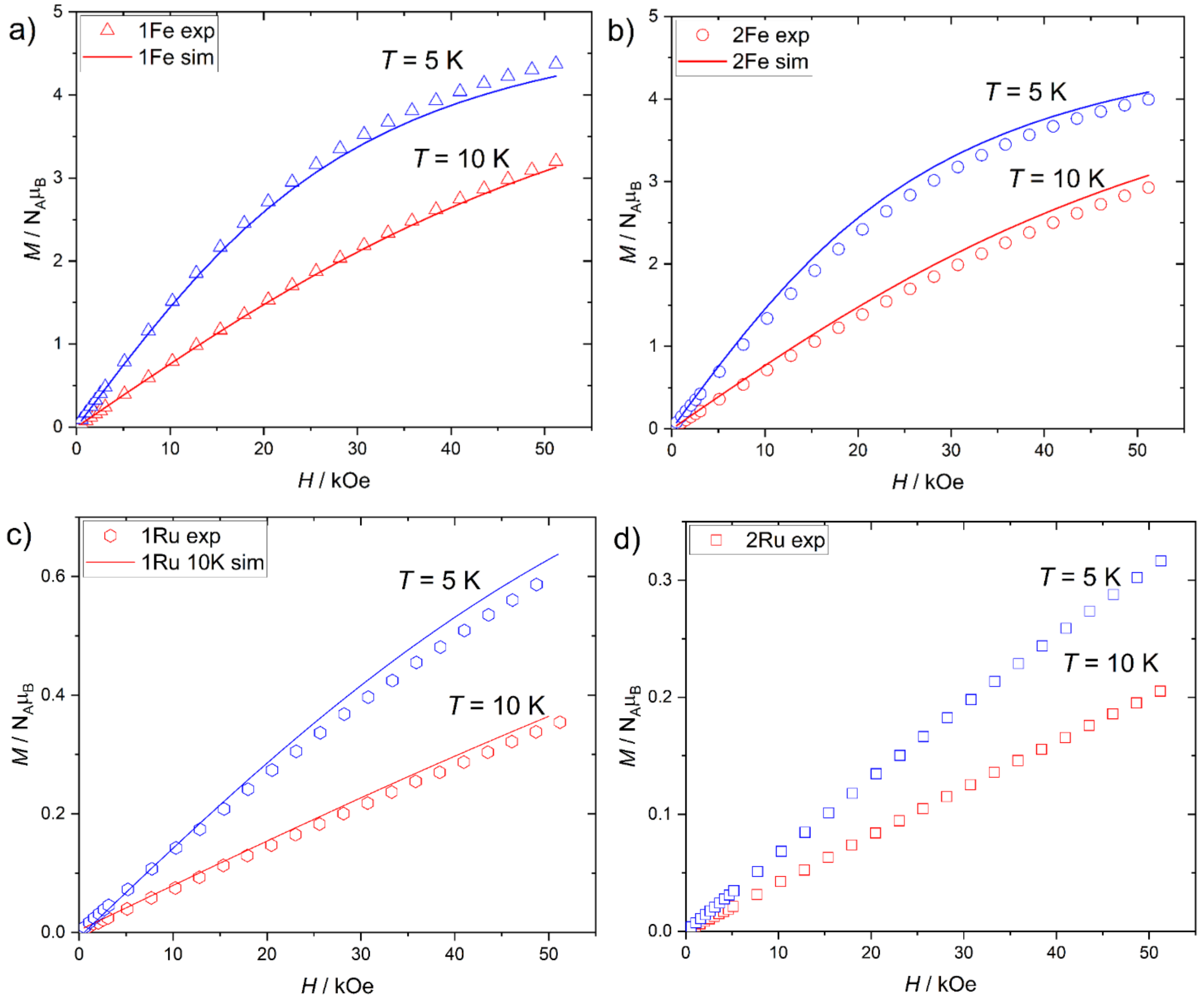

Figure SI-12. Magnetization curves (a to d for **1Fe, 2Fe, 1Ru, 2Ru**, respectively) recorded at *T* = 5 K (blue) and 10 K (red). Symbols represent experimental points and lines are the best fit. Data are reported per mole of metal ion.

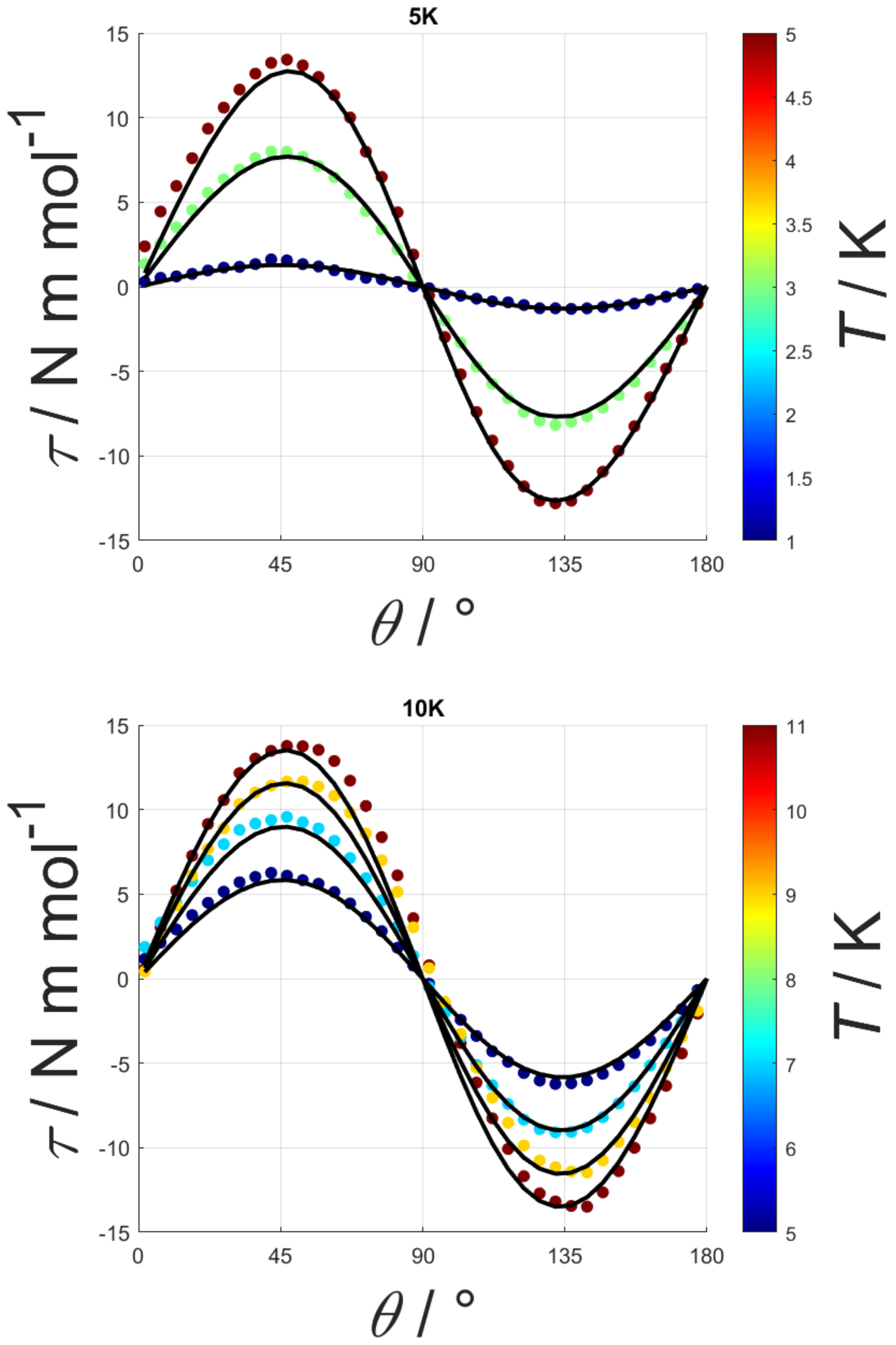

Figure SI-13. Angular dependence of the torque for **1Fe** for Rotation 1 measured at B = 1, 2, 3, 5 T (from blue to red) at T = 5 and 10 K. At the beginning of the clockwise rotation around the *a* crystallographic axis, the magnetic field was along the *c* crystallographic axis.

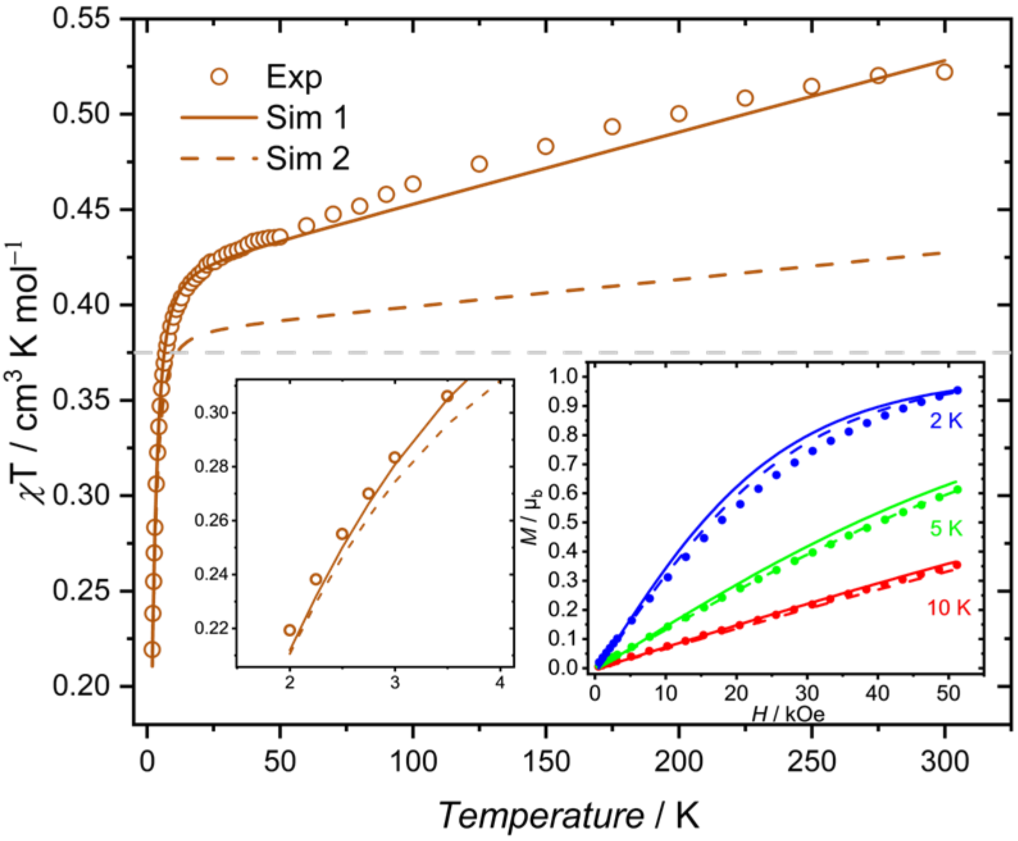

Figure SI-14. Experimental and simulated data for **1Ru**. χT vs T has been collected at H = 50 kOe to amplify the weak signal observed at high temperature. In the inset on the left, a magnification of χT vs T at low temperature is reported. In the inset on the right, the magnetization curves recorded at three different temperatures are reported. In all the graphs, points represent experimental data, continuous lines represent Simulation 1 (C_2^0 = 500 cm^{-1} and κ = 0.60, see main), and the dashed lines represent Simulation 2 (κ = 0.34 and C_0^2 = 1900 cm^{-1}, see main). The gray dashed line is the Curie constant value for an S = 1/2 system with g = 2.

References

1. A. J. Blake, G. A. Heath, G. Smith, L. J. Yellowlees and D. W. A. Sharp, *Acta Crystallographica Section C*, 1988, **44**, 1836-1838.
2. W. L. Armarego, *Purification of laboratory chemicals*, Butterworth-Heinemann, 2017.
3. S. Ito, F. J. White, E. Okunishi, Y. Aoyama, A. Yamano, H. Sato, J. D. Ferrara, M. Jasnowski and M. Meyer, *CrystEngComm*, 2021, **23**, 8622-8630.
4. G. Sheldrick, *Acta Crystallographica Section A*, 2015, **71**, 3-8.
5. G. Sheldrick, *Acta Crystallographica Section C*, 2015, **71**, 3-8.
6. O. V. Dolomanov, L. J. Bourhis, R. J. Gildea, J. A. K. Howard and H. Puschmann, *Journal of applied crystallography*, 2009, **42**, 339-341.
7. C. B. Hubschle, G. M. Sheldrick and B. Dittrich, *Journal of applied crystallography*, 2011, **44**, 1281-1284.
8. A. Saha, S. S. Nia and J. A. Rodríguez, *Chemical reviews*, 2022, **122**, 13883-13914.
9. L.-M. Peng, *Micron*, 1999, **30**, 625-648.
10. F. H. Allen and I. J. Bruno, *Structural Science*, 2010, **66**, 380-386.
11. A. L. Pellegrino, C. Mezzalira, F. Mazzer, L. Cadi Tazi, A. Caneschi, D. Gatteschi, I. L. Fragalà, A. Speghini, L. Sorace and G. Malandrino, *Inorganica Chimica Acta*, 2022, **535**.
12. S. Stoll and A. Schweiger, *J. Magn. Reson.*, 2006, **178**, 42-55.
13. C. Adamo and V. Barone, *J. Chem. Phys.*, 1999, **110**, 6158-6170.
14. S. Grimme, J. Antony, S. Ehrlich and H. Krieg, *The Journal of Chemical Physics*, 2010, **132**.
15. F. Weigend and R. Ahlrichs, *Phys. Chem. Chem. Phys.*, 2005, **7**, 3297-3305.
16. S. Schmitt, P. Jost and C. van Wüllen, *The Journal of Chemical Physics*, 2011, **134**.
17. F. Neese, *The Journal of Chemical Physics*, 2005, **122**.
18. F. Dwyer and A. Sargeson, *Journal of the American Chemical Society*

1955, **77**, 1285-1285.

19. A. Raza, L. Chelazzi, S. Ciattini, L. Sorace and M. Perfetti, *Inorg. Chem.*, 2024, **63**, 17198-17207.
20. D. N. Rainer, 2025, DOI: 10.5281/zenodo.17328491.
